# Glyco-Conjugation of Thiosemicarbazone Copper(II) Complexes Enhances Selectivity toward Cancer Cells Overexpressing GLUT1

**DOI:** 10.1021/acsomega.6c02319

**Published:** 2026-05-29

**Authors:** Francesca Miglioli, Alessio Zavaroni, Cristina Marzano, Diego Montagner, Mauro Carcelli, Valentina Gandin, Dominga Rogolino

**Affiliations:** † Department of Chemistry, Life Sciences, Environmental Sustainability, 9370 University of Parma, Parco Area delle Scienze 11/A, 43124 Parma, Italy; ‡ Department of Pharmaceutical and Pharmacological Sciences, University of Padova, via F. Marzolo 5, 35131 Padova, Italy; § Department of Chemistry, 8798 Maynooth University, Maynooth W23 F2H6, Ireland

## Abstract

To overcome the limitations of conventional chemotherapy, specifically poor selectivity and systemic toxicity, this study explores a targeted approach leveraging the Warburg effect. We developed a series of novel copper­(II) glucose-conjugated thiosemicarbazones (C1–C4), rationally designed to enhance water solubility and achieve good selectivity toward cancer cells. *In vitro* evaluations across a panel of human cancer cell lines demonstrated that free ligands were largely inactive, whereas copper­(II) coordination significantly enhanced cytotoxicity. A strong linear correlation was observed between GLUT1 expression levels and cytotoxic potency, suggesting a possible involvement of GLUT-mediated uptake. Furthermore, when tested against HEK-293 cells, these complexes displayed a promising degree of selectivity, with selectivity indices (SI) up to four times higher than that of cisplatin. Mechanistic investigations indicated efficient intracellular accumulation together with inhibition of Protein Disulfide Isomerase (PDI). Morphological analysis via TEM and confocal microscopy in PSN-1 pancreatic cancer cells revealed features consistent with paraptosis rather than classical apoptosis. These findings suggest that metal-based glyco-conjugation is a highly effective strategy for developing selective, potent, and water-soluble anticancer agents capable of bypassing traditional drug resistance pathways.

## Graphical abstract

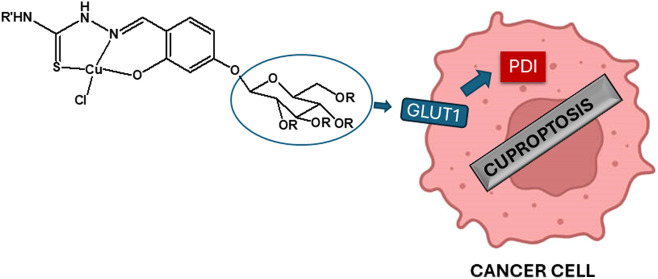

## 1 Introduction

Even though medicine has achieved significant milestones in the fight against cancer, obviously there is still the need to improve selectivity toward cancer cells and limiting side effects of clinical treatments. One promising strategy leverages the significant metabolic differences between healthy and cancer cells, offering a potential pathway to specifically target the last ones. The dysfunctional metabolism of cancer cells was first described by Otto Warburg, who observed that solid tumors avidly consume glucose compared to normal tissue.^1^ This abnormal energy consumption, known as the Warburg effect, results in the predominance of glycolysis over oxidative phosphorylation. Consequently, malignant cells exhibit high glucose requirements and overexpress glucose transporters (GLUTs) compared to healthy cells. Recognizing that deregulation of cellular energetics is a cancer hallmark and that tumor glycolysis could represent a promising target for clinical intervention, has driven researchers toward the development of new drugs. One strategy involves the development of inhibitors that target critical glycolytic enzymes.^2^ These inhibitors aim to disrupt the cancer cell primary energy-producing pathway, leading to a decrease in ATP and an inhibition of processes necessary for cell proliferation in a specific manner. Another strategy is glyco-conjugation, which involves attaching a sugar moiety or a glycomimetic substrate directly to a therapeutic agent.^3–6^ The overexpression of GLUTs in cancer cells could in fact be exploited to achieve site-specific delivery of cytotoxic compounds. The glucose moiety should be recognized by GLUTs and the chemical scaffold anchored to the glucose internalized as a whole. This could enhance the drug uptake in the tumor cells, improving its activity and selectivity toward cancer cells, avoiding the severe side-effects of a carpet-bombing chemotherapy. Notably, the insertion of a sugar moiety on the molecular backbone of the anticancer agents can also improve water solubility. Glufosfamide, initially reported by Wiessler and colleagues in 1995, was the first sugar conjugate designed as an anticancer compound exploiting the Warburg effect in its mode of action.^7^ Since then, many other examples have been described in the literature, supporting the idea that glycosylated derivatives could represent a promising alternative to the corresponding aglycones. These examples include various glyco-conjugates of taxoids (like docetaxel and paclitaxel),^8^ DNA alkylators (for example, chlorambucil and busulfan)^9,10^ or intercalators (such as doxorubicin).^6^

Over the last several decades, metal-based drugs have played a crucial role in cancer treatment. Cisplatin has been a milestone of clinical practice and since its discovery other platinum-based anticancer drugs have been approved, including oxaliplatin and carboplatin, while others are in clinical trials.^11,12^ Several Pt­(II)-glycoconjugated have been developed starting from the early 2000th, demonstrating high water-solubility and anticancer activity in xenograft models when compared to cisplatin.^13–15^ Experimental data support the idea that conjugates enter cancer cells, at least in part, through overexpressed glucose transporters.^16,17^ To a lesser extent, glyco-conjugation has been applied also to Pt­(IV) cytotoxic compounds, validating the efficacy of this pharmacological approach.^18–20^ Furthermore, metal-based glyco-conjugates involving various metals and ligands have been investigated and revealed promising cytotoxic activity.^21^

Thiosemicarbazones (TSCs) have deserved much attention for their biological properties, including the anticancer ones; notably, some of them (Triapine, COTI-2, and DpC) are currently in clinical trials.^22,23^ They have excellent metal-chelating properties, and when combined with metal ions, they become even more potent against cancer cells. The metal complexes can exert their cytotoxicity in different ways, for example, by inhibiting enzymes involved in DNA synthesis, disrupting iron or copper metabolism, and increasing oxidative stress, offering a valid alternative to platinum-based drugs and potentially leading to better selectivity and reduced side effects.^24–27^ Glycoconjugation has been recently applied in the design of disulfide-masked TSCs that are reductively activated in cells to form high-affinity iron chelators, targeting both elevated glucose consumption and iron metabolism of cancer cells.^28,29^

We have previously disclosed a series of 2,3-dihydroxy- and 2-hydroxy-3-methoxy-benzaldehyde TSCs and their copper­(II) complexes, endowed with *in vitro* activity in the low nanomolar range (Figure 1).^30^ These compounds potently inhibit Protein Disulfide Isomerase (PDI), a copper-binding protein, that is emerging as a new therapeutic target for cancer treatment.^31,32^ Despite their potency, further development was hampered by limited aqueous solubility. To overcome this drawback, we then designed water-soluble sulfonated TSCs analogues (Figure 1), which showed promising results in preliminary *in vitro* and *in vivo* evaluations.^33^

**Figure 1:**
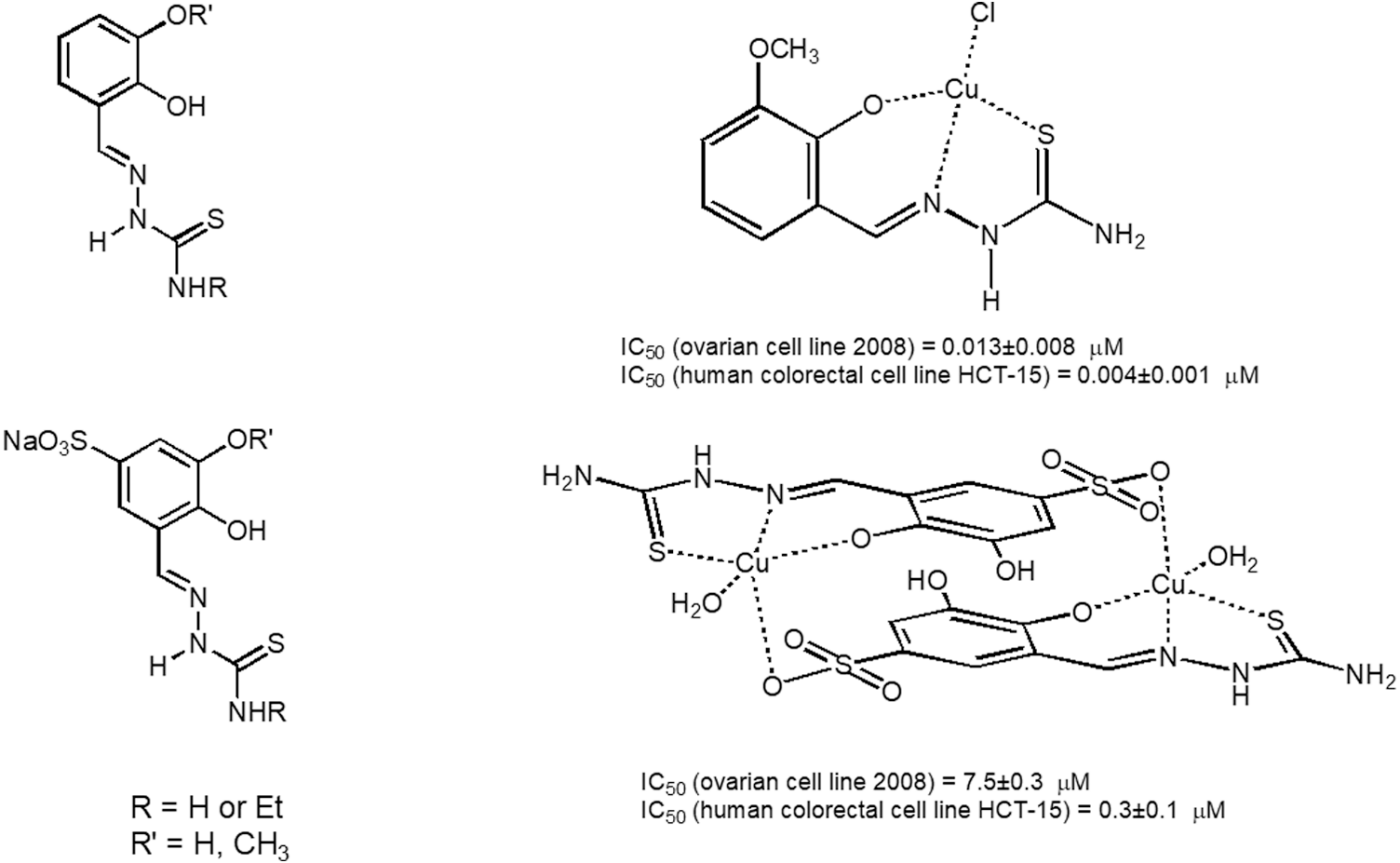
Scheme of some TSCs ligands and copper­(II) complexes previously investigated for their anticancer activity.

Starting from these considerations, here we present a new series of TSCs ligands obtained by O-glycosylation of the scaffold (Figure 2): two ligands are glucose-conjugated (**H**_**2**_ **L3** and **H**_**2**_ **L4**) and two are the parent acetylated derivatives (**H**_**2**_ **L1** and **H**_**2**_ **L2**). This modification was conceived as a dual strategy to enhance aqueous solubility while introducing a glucose moiety potentially exploitable for transporter-mediated uptake. The newly synthesized ligands **H**_**2**_ **L1-H**_**2**_ **L4** and their copper­(II) complexes **C1**-**C4** (Figure 2), were therefore designed to retain cytotoxic and PDI-inhibitory properties, while potentially achieving improved tumor selectivity through GLUT-dependent internalization. To note that acetylated carbohydrate derivatives have been often found to have higher anticancer activity than their deprotected counterparts, likely due to an increase in lipophilicity that facilitates diffusion through cell membranes.^34–36^

**Figure 2:**
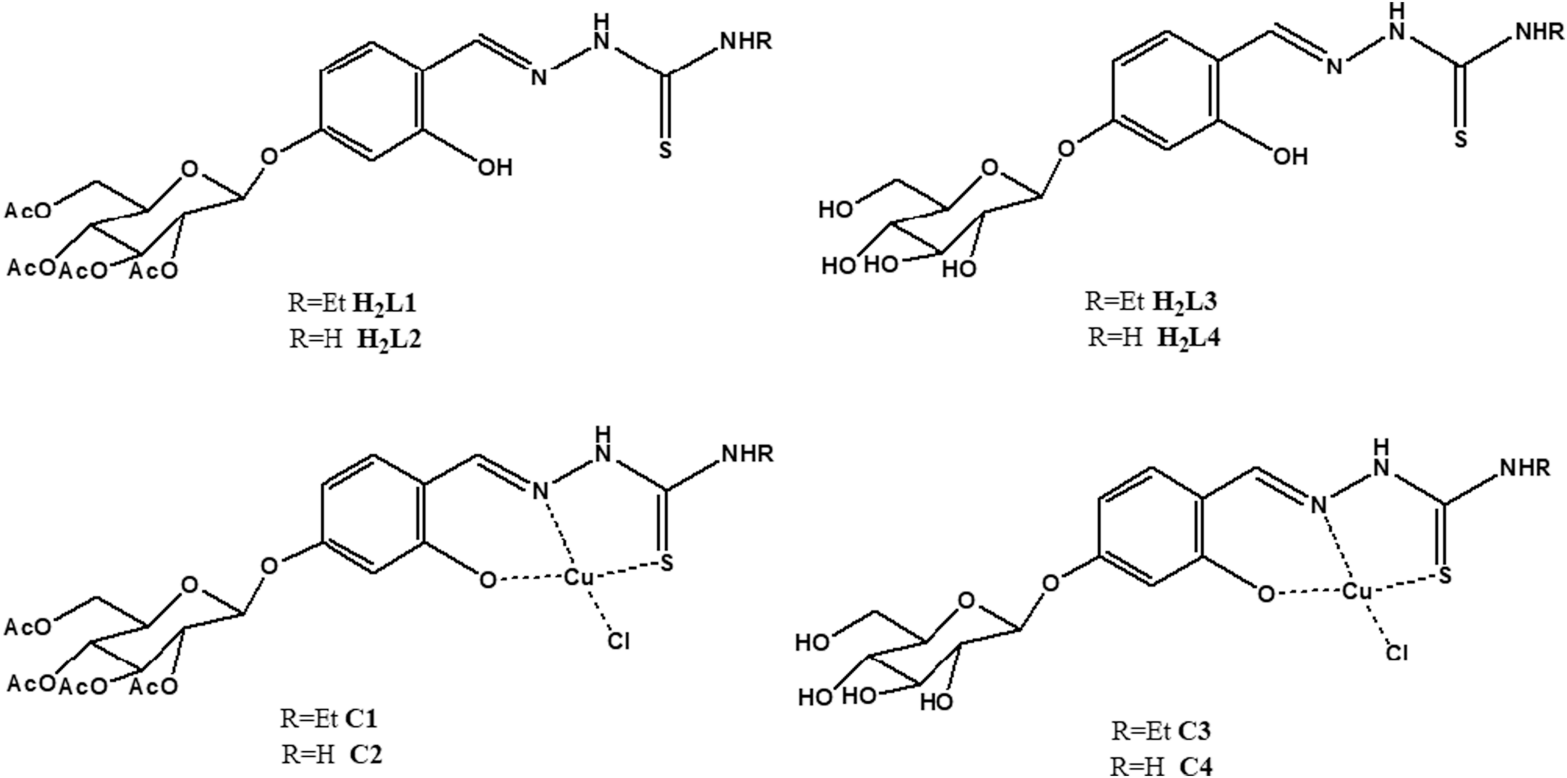
Scheme of the O-glycosylated TSCs ligands **H**_**2**_ **L1**-**H**_**2**_ **L4** and of the corresponding copper­(II) complexes **C1**-**C4**.

## 2 Results and Discussion

### 2.1 Synthesis

The glycosylated ligands **H**_**2**_ **L1-H**_**2**_ **L4** were obtained according to Scheme 1.

**Scheme 1:**
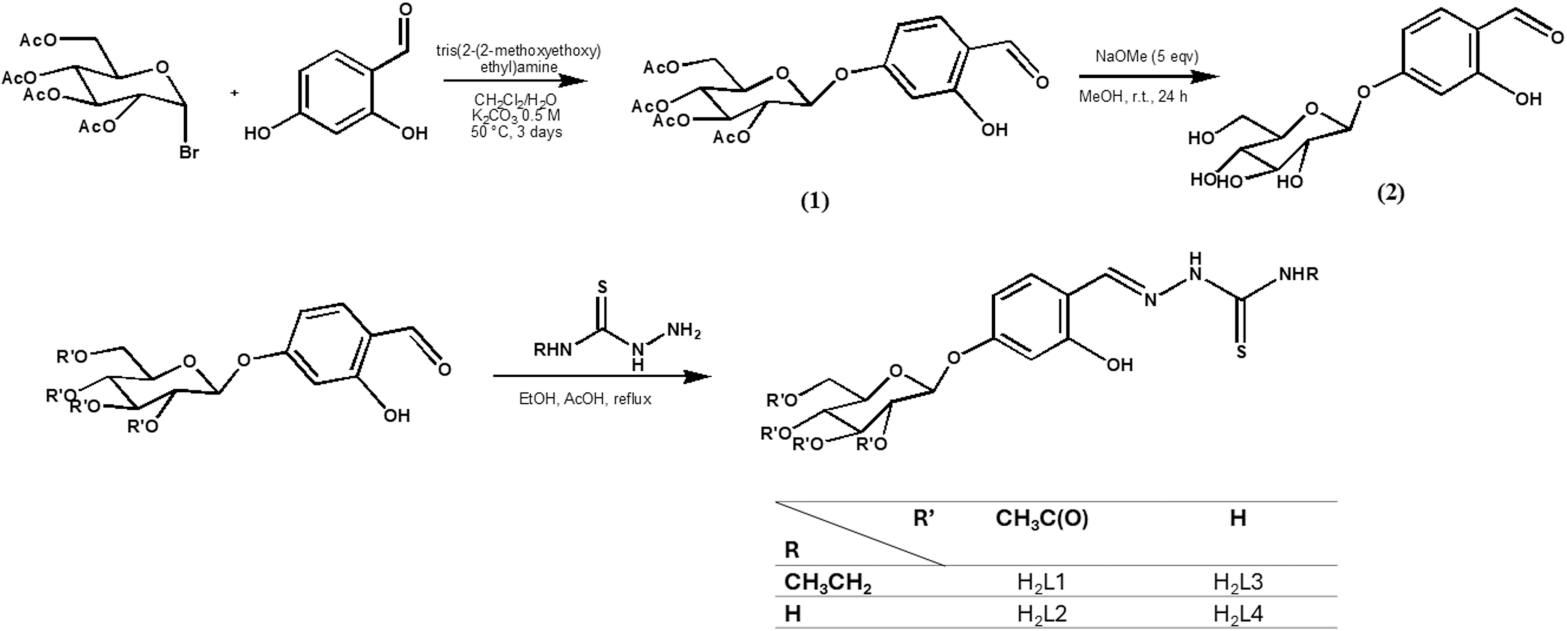
Synthetic Pathway for the Isolation of the O-Glycosylated TSCs Ligands **H**_**2**_ **L1**-**H**_**2**_ **L4**

2,4-dihydroxy benzaldehyde was reacted with a slight excess of acetobromo-α-d-glucose under phase-transfer conditions in a mixture of dichloromethane and aqueous K_2_CO_3_ solution using tris­[2-(2-methoxyethoxy)­ethyl]­amine as the phase-transfer agent.^37^ Compound **(1)** was obtained as a white solid (*Y* = 46%) after chromatographic purification (Scheme 1, Experimental section). Deacetylation of **(1)** was then accomplished by adding sodium methoxide in methanol, obtaining compound **(2)** as a solid (*Y* = 72%). The complete conversion was evident from the disappearance in the ^1^H NMR spectrum of (**2)** of the signals around 2 ppm, corresponding to the methyl protons of the acetyl groups. Additionally, the glucose CH signals exhibited a shift to lower ppm and an increase in multiplicity, which are characteristic of the deprotected sugar moiety (Figure S4). Moreover, in the IR spectrum of **(2)** the band at 1744 cm^–1^ relative to the stretching of the carbonyl groups of the acetyl moieties is absent (Figure S5). Condensation reaction of aldehydes **(1)** and **(2)** with thiosemicarbazide or ethyl thiosemicarbazide afforded the glyco-TSCs ligands **H**_**2**_ **L1-H**_**2**_ **L4** (Scheme 1). The successful outcome of the reaction was consistently confirmed across various analytical techniques (Figures S7–S22). For instance, the ^1^H NMR spectrum of **H**_**2**_ **L1-H**_**2**_ **L4** in DMSO-*d*_6_ clearly showed the disappearance of the aldehydic proton signal and the appearance of the iminic proton one at about δ 8.3 ppm. For all ligands, only the thione E-form appeared to be present in DMSO-*d*_6_ solution at room temperature. HRMS analysis provided further characterization of the ligands, with the observation of molecular ion peaks in positive ionization modes. The copper­(II) complexes **C1–C4** (Figure 2) with both acetylated and deprotected sugar moieties in the ligands are then synthesized by reacting one equivalent of CuCl_2_ with the corresponding ligand.^33^ The complexes were thoroughly characterized using elemental analysis, FT-IR, and HRMS. Comparison of the IR spectra of the complexes with those of their free ligands supports a ONS tridentate coordination of the ligands to the copper­(II) ions. For instance, the stretching O–H band around 3400 cm^–1^ disappears in the spectrum of **C2** (Figure S23), indicating the deprotonation of the corresponding ligand **H**_**2**_ **L2** hydroxyl group. Elemental analysis results are consistent with the [Cu­(HL)­Cl] stoichiometry, with different degrees of hydration depending on the complex: it is reasonable to suppose that the copper­(II) ion is coordinated by the monodeprotonated, tridentate TSC and a chloride ion completes its coordination. HRMS spectra further support the proposed structures, as molecular ions are visible in positive ionization mode for all the complexes, typically as sodium adducts (Figures S20, S22, S24 and S26). The stability of the copper­(II) complexes was evaluated by recording their UV spectra in solution (*C* ∼ 40–50 μM in 25 mM HEPES buffer, pH = 7.4 in H_2_O containing 0.9% NaCl) over 72 h; the final solutions of **C1** and **C2** contained 2% DMSO, as these complexes are not completely water-soluble. These conditions were chosen to simulate the solutions used for *in vitro* cell line tests. Figure S28 presents the UV spectra of the copper complexes. The UV spectra of **C2**, **C3** and **C4** did not change in 72 h, while a change in the absorbance values was observed for **C1**, likely because of its low solubility under these conditions.

### 2.2 Cell Culture and Mechanistic Studies

The newly synthesized copper­(II) complexes **C1–C4**, along with the ligands **H**_**2**_ **L1-H**_**2**_ **L4** were evaluated for their cytotoxic effects using two-dimensional (2D) cell culture models. The compounds were screened against a panel of human cancer cell lines, including colon (HCT-15), breast (MCF-7), pancreatic (PSN-1), thyroid (B-CPAP), and ovarian (2008, and their cisplatin-resistant subline, C13*) models, selected for their different degree of GLUT expression. Cisplatin was used as the reference metallodrug under identical experimental conditions. Cytotoxicity was assessed using the SRB assay following 72 h of exposure, and IC_50_ values are summarized in Table 1.

**Table 1:** IC_50_ Values for Cells (3–8 × 10^3^ Cell/Well) Treated for 72 h with Tested Compounds^a^

IC_50_ (μM) ± S.D.							
Compound	HCT-15	MCF-7	PSN-1	B-CPAP	2008	C13* (R.F.)	HEK-293
**C1**	16.2 ± 3.1	25.4 ± 2.8	4.3 ± 1.0	6.4 ± 1.2	15.4 ± 2.1	14.1 ± 1.2 (0.9)	>50
**C2**	18.5 ± 2.4	23.2 ± 3.6	1.2 ± 0.2	3.9 ± 1.0	17.5 ± 2.3	15.6 ± 1.5 (0.9)	>50
**C3**	19.1 ± 3.5	23.3 ± 1.6	5.1 ± 0.5	7.3 ± 1.2	19.4 ± 1.8	17.2 ± 2.1 (0.9)	>50
**C4**	9.2 ± 1.2	12.5 ± 1.5	0.1 ± 0.03	0.8 ± 0.2	9.0 ± 1.1	8.3 ± 0.9 (0.9)	45.5 ± 2.7
**H**_**2**_ **L1**	>50	>50	31.3 ± 3.1	42.4 ± 1.4	>50	>50	n.t
**H**_**2**_ **L2**	>50	>50	29.7 ± 1.8	40.3 ± 2.3	>50	>50	n.t
**H**_**2**_ **L3**	>50	>50	33.5 ± 2.6	43.7 ± 3.1	>50	>50	n.t.
**H**_**2**_ **L4**	>50	>50	37.6 ± 3.2	35.6 ± 2.3	>50	>50	n.t.
**Cisplatin**	10.5 ± 1.9	16.5 ± 3.1	16.4 ± 2.4	13.2 ± 2.1	1.8 ± 0.4	23.6 ± 1.9	19.6 ± 3.2

a Cell viability was estimated by means of the SRB test. The IC_50_ values were calculated by a four-parameter (4-PL) logistic model (with a significance level of *p* < 0.05). Data are presented as the mean ± standard deviation (SD) from five independent measurements. Resistance Factor = IC_50_ resistant/IC_50_ parental cells. R.F. = resistance factor.

The free ligands **H**_**2**_ **L1**–**H**_**2**_ **L4** displayed overall low cytotoxic activity, with only modest effects in PSN-1 and B-CPAP cells. Upon coordination to copper­(II), a marked increase in cytotoxicity was observed for all complexes, indicating that metal coordination plays a key role in modulating biological activity. Across the series, the copper­(II) complexes showed low micromolar potency, with minimal differences between the acetylated **C1–C2** and deacetylated **C3** derivatives. However, the unacetylated complex **C4** emerged as the most active, with a mean IC_50_ of 6.3 μM, approximately 2-fold lower than that observed for the reference metallodrug cisplatin (11.7 μM). Notably, cytotoxic responses varied substantially across the cell panel and appeared to be cell line-dependent. The highest sensitivity was observed in PSN-1 and BCPAP cells, which also displayed the most elevated GLUT1 expression levels (Figure 3A).

**Figure 3:**
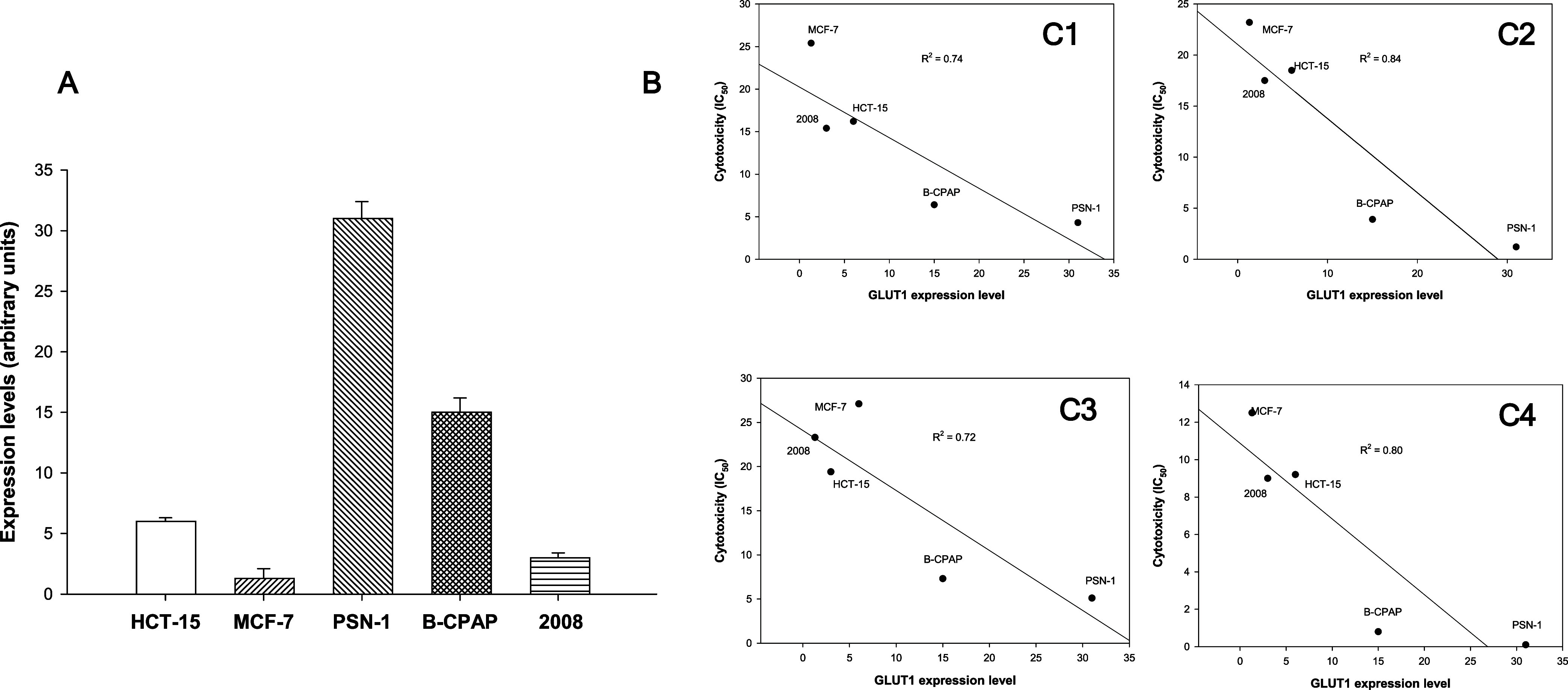
GLUT1 levels estimated by ELISA in human cancer cells (A) and correlation with cytotoxicity in 2D by a simple linear regression model (*R* ^2^) (B). Data are presented as the mean ± standard deviation (SD) from three independent measurements.

A correlation between GLUT1 levels and cytotoxicity potency was observed with *R* ^2^ values ranging from 0.72 to 0.84 (Figure 3B), suggesting a possible contribution of transporter-mediated uptake, although additional studies are required to confirm this hypothesis.

The cytotoxicity of the novel copper­(II) compounds was also investigated in a cisplatin-resistant subline (C13* cells) where all complexes retained comparable potency relative to the parental cisplatin-sensitive 2008 cells. The resulting resistance factor (R.F., Table 1) values close to unity indicate a lack of cross-resistance.

The antiproliferative activity of the novel glycosylated copper complexes was also evaluated in nontumorigenic human HEK-293 embryonic kidney cells (Table 1) to provide a preliminarily assessment of their selectivity. Complexes **C1–C3** exhibited low cytotoxic activity in this cell model, while the most active compound **C4** showed a selectivity index (SI = the quotient of the mean IC_50_ in nontumorigenic cells divided by the mean IC_50_ in malignant cells) of 7.2, about 4-fold higher than that detected with the reference platinum drug cisplatin (SI= 1.7), suggesting a promising but preliminary degree of selectivity.

Given the pronounced cytotoxic effects observed in 2D monolayer cultures, the activity of the new glycosylated complexes was further evaluated using established three-dimensional (3D) cell culture models. Three-dimensional spheroid systems are widely recognized to more closely mimic the *in vivo* tumor microenvironment, encompassing key physio-pathological features such as gene expression, cell–cell interactions, and metabolic profiles, as well as drug-related processes including penetration, retention, and intracellular trafficking, thereby providing improved translational relevance. Table 2 reports the IC_50_ values obtained after treatment of human ovarian (2008) and pancreatic (PSN-1) cancer–derived 3D spheroids with complexes **C1–C4** and the corresponding ligands **H**_**2**_ **L1-H**_**2**_ **L4**, with cisplatin included as the reference drug.

**Table 2:** IC_50_ Values for Spheroids Treated for 72 h with Increasing Concentrations of Tested Compounds^a^

IC_50_ (μM) ± S.D.		
Compound	PSN-1	2008
**C1**	47.5 ± 2.6	70.4 ± 3.0
**C2**	34.6 ± 3.1	58.5 ± 4.5
**C3**	62.3 ± 4.3	77.4 ± 3.8
**C4**	21.6 ± 3.3	46.9 ± 2.1
**H**_**2**_ **L1**	>100	>100
**H**_**2**_ **L2**	>100	>100
**H**_**2**_ **L3**	>100	>100
**H**_**2**_ **L4**	>100	>100
**Cisplatin**	87.4 ± 6.7	19.4 ± 3.3

a Cytotoxicity was assessed by means of a modified APH assay. IC_50_ values were calculated from the dose–survival curves by the 4-PL model (with a significance level of *p* < 0.05). Data are presented as the mean ± standard deviation (SD) from five independent measurements.

The collected data indicate that the copper­(II) complexes were substantially more cytotoxic than the corresponding free ligands and showed enhanced potency toward the highly GLUT1-expressing PSN-1 cells, further supporting a possible link between biological activity and cellular type. Compound **C4** was confirmed as the most active derivative, displaying an IC_50_ value approximately 4-fold lower than that of cisplatin against PSN-1 cells.

To investigate whether cytotoxicity correlated with intracellular copper accumulation, uptake studies were carried out in PSN-1 cells. Cells were treated for 24 or 36 h with the tested complexes and intracellular copper content was quantified by graphite furnace atomic absorption spectrometry (GF-AAS) and expressed as ng of metal per 10^6^ cells (Figure 4A).

**Figure 4:**
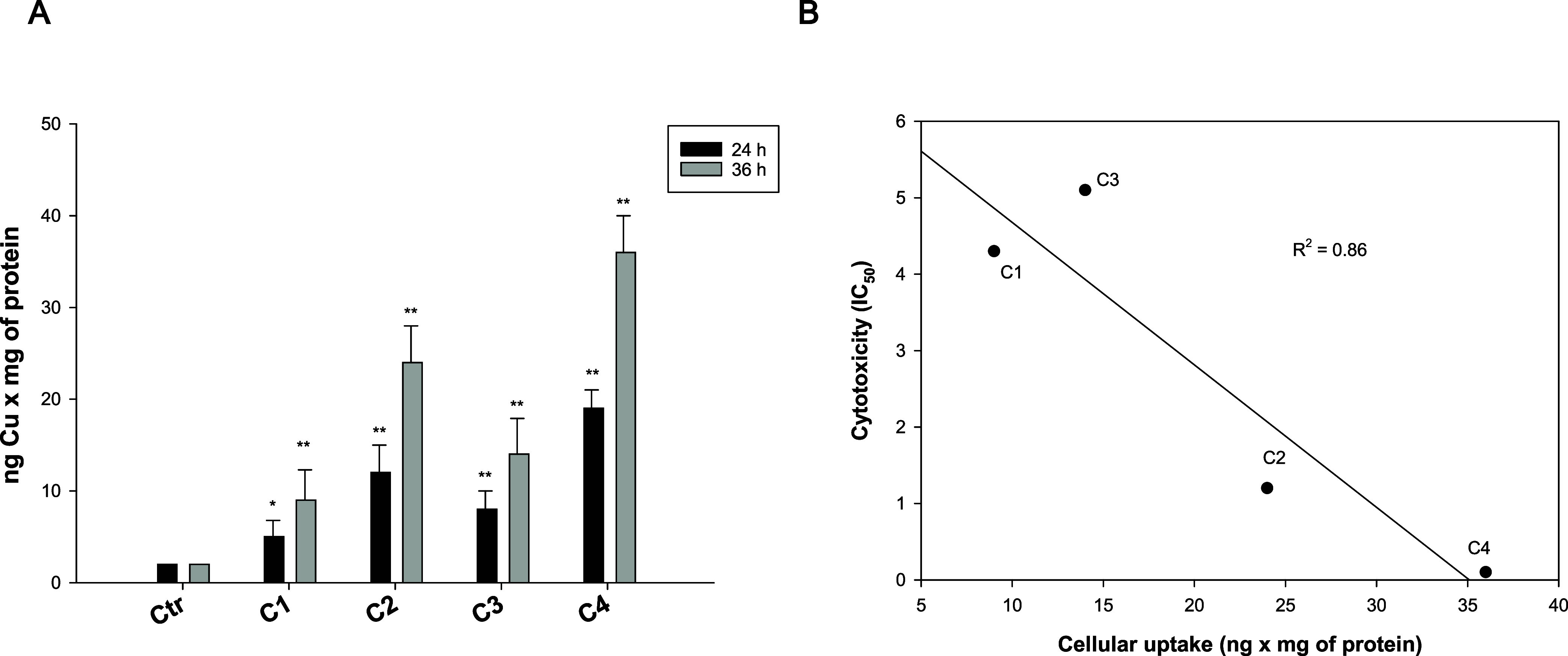
Cellular uptake. Intracellular accumulation of copper complexes **C1**-**C4** (A), and correlation between copper content and cytotoxicity by a simple linear regression model (*R* ^2^) (B). PSN-1 cells were incubated with copper complexes for 24 and 36 h, and cellular copper content was detected by GF-AAS analysis. Data are presented as the mean ± standard deviation (SD) from three independent measurements. *p < 0.05; **p < 0.01 compared with the control.

All copper­(II) compounds efficiently accumulated within pancreatic cancer cells, with **C4** yielding the highest intracellular copper levels. When uptake data were compared with cytotoxicity results in PSN-1 cells (Figure 4B), a qualitative correlation between intracellular internalization (at 36 h) and cytotoxic potency was observed.

Over the past three decades, numerous mechanistic investigations on copper­(II) complexes have identified multiple molecular targets. Among them, protein disulfide isomerase (PDI) was recently highlighted as a key target for several copper­(I) and copper­(II) derivatives [30,33]. In light of this, we evaluated the ability of **C1–C4** to behave as PDI inhibitors. The PDI enzyme was treated with increasing concentrations (1–100 μM) of the complexes, and inhibition was quantified using a colorimetric assay (Proteostat PDI kit). All copper derivatives **C1–C4** elicited IC_50_ values in the micromolar range (Table 3), with **C4** showing the strongest inhibitory activity (IC_50_ = 15.5 ± 5.7 μM). These values were markedly lower than that of bacitracin (IC_50_ = 570 ± 12 μM), used as a reference PDI inhibitor.

**Table 3:** PDI Inhibition Induced by **C1–C4** Was Measured by Proteostat PDI Assay Kit^a^

IC_50_ (μM) ± S.D.	
**C1**	23.5 ± 4.2
**C2**	37.2 ± 4.4
**C3**	22.5 ± 5.1
**C4**	15.5 ± 5.7
**Bacitracin**	570 ± 12

a The PDI inhibitor Bacitracin was used as a reference PDI inhibitor. IC_50_ values were calculated from the dose–survival curves by the 4-PL model (with a significance level of *p* < 0.05). Data are presented as the mean ± standard deviation (SD) from five independent measurements.

It has been previously demonstrated that copper complexes induce endoplasmic reticulum (ER) stress and trigger nonapoptotic forms of programmed cell death, including paraptosis-like mechanisms.^38^ More recently, copper-dependent cell death pathways, collectively termed cuproptosis, have been described.^39^ In order to characterize the cellular morphological changes induced by the newly developed copper­(II) compounds, PSN-1 pancreatic cancer cells were treated with complex **C4**, chosen as the most representative, and analyzed by transmission electron microscopy (TEM) (Figure 5A) or confocal microscopy (Figure 5B). Cells treated with **C4** showed the nucleus with a clearly defined ultrastructure, and regularly distributed chromatin, whereas mitochondria appeared enlarged (swelled) and displayed altered internal structures. Striking morphological changes, including ER swelling and cytoplasmic vacuolization, previously characterized as hallmarks of paraptotic-like cell death,^34^ were distinctly noticed (Figure 5A). Consistently, **C4**-treated cells stained with Hoechst 33342 did not show nuclear DNA condensation or apoptotic body formation, supporting the activation of a cancer cell death pathways distinct from classical apoptosis (Figure 5B). By contrast, classical hallmarks of apoptosis (e.g., brightly stained nuclei, chromatin condensation and fragmentation) were clearly evident in PSN-1 cells treated with the reference metallodrug cisplatin.

**Figure 5:**
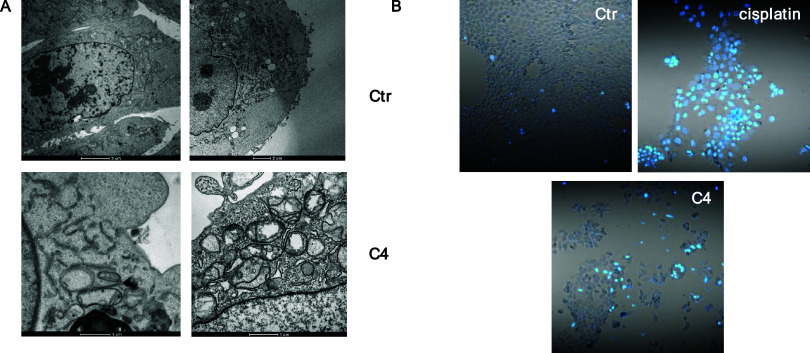
Morphological analysis. (A) TEM analysis of PSN-1 cancer cells untreated (ctr) or treated for 24 h with IC_50_ of complex (**C4**) (B) Hoechst staining of PSN-1 cells untreated (ctr) or incubated for 48 h with IC_50_ doses of cisplatin or complexes (**C4**) (10× magnification).

## 3 Conclusions

The **H**_**2**_ **L1**-**H**_**2**_ **L4** thiosemicarbazone ligands, obtained through O-glycosylation of the salicylaldehyde chelating scaffold, showed modest yet selective cytotoxic activity in GLUT1-overexpressing cell lines (PSN-1 and B-CPAP), while remaining largely inactive in HCT-15, MCF-7 and 2008 cells. These findings suggest that glycosylation may contribute to transporter associate selectivity. Coordination to copper­(II) markedly altered the biological profile of the ligands. The resulting **C1**-**C4** complexes exhibited cytotoxic potencies comparable to or exceeding that of cisplatin and retained preferential activity in GLUT-overexpressing models, with IC_50_ values in the low micromolar range for PSN-1 and B-CPAP cells. Importantly, cytotoxicity was maintained in 3D spheroid systems, where IC_50_ values were significantly lower than those of cisplatin and remained more pronounced in PSN-1 compared to 2008 spheroids. Moreover, **C1**-**C4** displayed negligible cytotoxicity toward nontumorigenic HEK293 cells, suggesting a promising, albeit preliminary, selectivity profile.

Beyond cytotoxic potency, metal complexes have long attracted interest, due to their distinctive mechanistic mode of action. We have demonstrated that **C1**-**C4** complexes inhibit the activity of PDI, an emerging vulnerability in cancer cells [32], with IC_50_ values in the micromolar range. Among the series, **C4** proved to be the most active complex, combining the strongest PDI inhibition (IC_50_ = 15.5 ± 5.7 μM) with the highest cytotoxic effects in PSN-1 (IC_50_ = 0.1 ± 0.03 μM) and B-CPAP (IC_50_ = 0.8 ± 0.2 μM) cells. While this observation may indicate a possible interplay between intracellular copper accumulation, PDI inhibition, and tumor-selective cytotoxicity, further studies will be required to establish a causal relationship. Ultrastructural analyses by transmission electron microscopy and confocal imaging in PSN-1 cells revealed that **C4** induces a profound mitochondrial swelling and ER dilation in the absence of nuclear condensation, features consistent with a non-apoptotic, paraptosis like cell death mechanism. Collectively, these findings suggest that glycosylated copper­(II) thiosemicarbazones **C1**-**C4** may represent a promising class of metal-based agents capable of targeting metabolic and proteostatic vulnerabilities in cancer cells. Although a detailed structure–activity relationship cannot be unambiguously established, due to the interplay of multiple structural and physicochemical parameters, the present findings are consistent with previous reports on sugar-conjugated metal-binding systems,^40^ where metal coordination and glycosylation were shown to act in a concerted manner in modulating biological properties. Given the multivariate nature of the system, further optimization of the delivery profile through conjugation with other sugars or biopolymers—aimed at achieving superior pharmacokinetic properties and enhanced selectivity—represents a key direction for future research.

Even if further mechanistic and biological investigations will be necessary to fully elucidate their mode of action and therapeutic potential, the targeting of metabolic transport, together with intracellular copper-dependent stress responses, supports their potential as next-generation anticancer agents capable of overcoming resistance to apoptosis.

## 4 Experimental Section

### 4.1 Chemical Synthesis

All reagents and solvents were purchased from commercial sources (Merck Chemicals) and used without any further purification.

NMR spectra were recorded at 25 °C on a Bruker Advance spectrometer operating at 400 or 500 MHz for ^1^H and at 101 MHz for ^13^C nuclei, respectively. All chemical shifts are expressed in ppm. Infrared (IR) spectra were recorded in the region 4000–400 cm^–1^ on a PerkinElmer spectrometer, samples were run using ATR. HRMS spectra were acquired with a Bruker MaXis HD ESI-QTOF mass spectrometer coupled to a Thermo Scientific Dionex Ultra High Performance Liquid Chromatography (UPLC) unit. Electrospray mass spectral analyses (ESI-MS) were performed with a Waters electrospray ionization (ESI) time-of-flight Micromass 4LCZ spectrometer. Samples were dissolved in methanol.

Elemental analyses (carbon, hydrogen, nitrogen and sulfur) were performed with a Thermo Fisher FlashSmart CHNS/O analyzer with gas-chromatographic separation.

#### 4.1.1 4-(2′,3′,4′,6′-Tetra-*O*-acetyl-β-d-glucopyranosyloxy)-2-hydroxybenzaldehyde (**1**)

2,4-dihydroxy benzaldehyde (0.896 g, 6.48 mmol, 1 equiv) was dissolved in an aqueous K_2_CO_3_ solution (0.5 M, 11 mL) with tris­(2-(2-methoxyethoxy)­ethyl)­amine (3.11 mL, 9.73 mmol, 1.5 equiv). A solution of α-d-acetobrome glucose (4 g, 9.73 mmol, 1.5 equiv) in dichloromethane (11 mL) was added. The reaction mixture was left stirring at 47 °C for 3 days, then cooled to r.t. and 40 mL of water were added. The organic phase was separated, and the aqueous phase was extracted 3 times with dichloromethane. The combined organic phases were washed with HCl 1 M (1×) and with water (2×), then dried over Na_2_SO_4_, filtered and concentrated *in vacuum*. The residue was purified by silica chromatographic column (petroleum ether/ethyl acetate 65/35) obtaining **(1)** as a white solid (*Y* = 46%). IR (cm^–1^): ν­(C=O) 1745, 1660. ^1^H NMR (400 MHz, CDCl_3_) δ­(ppm): 11.39 (s, 1H); 9.80 (s, 1H); 7.50 (d, *J* = 8.6 Hz, 1H); 6.61 (dd, *J* = 8.6, 2.3 Hz, 1H); 6.57 (d, *J* = 2.3 Hz, 1H); 5.30 (m, 2H); 5.15 (m, 2H); 4.28 (dd, *J* = 12.3, 6.0 Hz, 1H); 4.18 (dd, *J* = 12.3, 2.4 Hz, 1H); 3.92 (m, 1H); 2.10 (s, 3H); 2.06 (m, 6H,); 2.04 (s, 3H). ^13^C­{^1^H} NMR (101 MHz; CDCl_3_) δ­(ppm): 194.8, 170.6, 170.2, 169.4, 169.2, 164.0, 163.2, 135.4, 116.7, 109.7, 103.6, 97.7, 72.5, 72.4, 70.9, 68.1, 61.9, 20.0. ESI-MS (positive ions, *m*/*z*): 491 [M + Na]^+^. Anal. Calcd. for C_21_H_24_O_12_: C 53.85%, H 5.16%. Found: C 54.47%, H 5.23%.

#### 4.1.2 4-(2′,3′,4′,6′-Tetra-hydroxy-β-d-glucopyranosyloxy)-2-hydroxybenzaldehyde (**2**)

0.520 g of **(1)** (1.11 mmol) were suspended in 10 mL of methanol and added of 5 equiv of sodium methoxide (0.3 g, 5.6 mmol) dissolved in 5 mL of methanol. The solution turned from white to red and it was left under stirring at r.t. overnight. A proton exchange resin (previously activated by sequential washing with HCl 2M, methanol/water and a final rinse with methanol) was added to the solution and after few minutes the pH was checked to be around 5. The resin was eliminated by filtration and the crude purified by chromatographic column (SiO_2_; dichloromethane/methanol 4:1), obtaining a pink solid (*Y* = 72%). IR (cm^–1^): ν­(OH) 3481, 3262; ν­(C=O) 1631. ^1^H NMR (400 MHz, DMSO-d_6_) δ­(ppm): 10.96 (s br, 1H); 10.04 (s, 1H), 7.62 (d, *J* = 8.7 Hz, 1H); 6.63 (dd, *J* = 8.7, 2.3 Hz, 1H); 6.58 (d, *J* = 2.2 Hz, 1H); 5.38 (d, *J* = 4.9 Hz, 1H); 5.13 (d, *J* = 4.6 Hz, 1H); 5.05 (d, *J* = 5.3 Hz, 1H); 4.95 (d, *J* = 7.4 Hz, 1H); 4.57 (t, *J* = 5.6 Hz, 1H); 3.69 (d, *J* = 12.1 Hz, 1H); 3.49 (m, 1H); 3.15–3.24 (m, 3H). ^13^C­{^1^H} NMR (101 MHz; DMSO-d_6_) δ­(ppm): 191.04; 163.79; 162.68; 131.93; 117.11; 108.51; 103.36; 99.71; 77.17; 76.46; 73.09; 69.46; 60.51. ESI-MS (positive ions, *m*/*z*): 323­[M + Na]^+^. Anal. Calcd. for C_13_H_16_O_8_: C 52.00%, H 5.37%. Found: C 51.74%, H 5.48%.

#### 4.1.3 4-(2′,3′,4′,6′-Tetra-*O*-acetyl-β-d-glucopyranosyloxy)-2- hydroxybenzaldehyde-4-ethyl-3- thiosemicarbazone (**H**_**2**_ **L1**)

**(1)** (0.250g, 0.534 mmol, 1 equiv) was dissolved at reflux in ethanol (4 mL), then ethylthiosemicarbazide (64 mg, 0.534 mmol, 1 equiv) and few drops of CH_3_COOH were added. The reaction mixture was left stirring at reflux for 4 h. After that time, the reaction was cooled to 0 °C, filtered and washed with cold ethanol, obtaining **H**_**2**_ **L1** as a white solid (*Y* = 85%). IR (ATR, cm^–1^): ν­(OH) = 3354; ν­(NH) = 3194, 2967; ν­(C=O) = 1746; ν­(C=N) = 1555; ν­(C=S) = 1032, 802. ^1^H NMR (400 MHz, DMSO-d_6_) δ­(ppm): 11.29 (s, 1H), 10.12 (b, 1H), 8.43 (t, *J* = 5.9 Hz, 1H), 8.28 (s, 1H), 7.89 (d, *J* = 8.9 Hz, 1H), 6.50 (s, 2H), 5.58 (d, *J* = 8.0 Hz, 1H), 5.47 (t, *J* = 9.6 Hz, 1H), 5.04 (m, 2H), 4.23 (m, 2H), 4.08 (d, 1H), 3.57 (m, 2H), 2.02 (s, 9H), 1.98 (s, 3H), 1.14 (t, *J* = 7.1 Hz, 3H). ^13^C­{^1^H} NMR (101 MHz; DMSO-d_6_) δ­(ppm): 177.5, 170.7, 170.2, 169.4, 169.2,160.0, 159.2, 147.8, 135.4, 133.0, 112.2, 109.5, 104.3, 98.1, 97.7, 72.5, 72.2, 70.9, 68.2, 61.9, 20.7, 20.6, 20.5. HRMS (positive ions, *m*/*z*): [M+Na]^+^ = 592.1567. Anal. Calcd for C_24_H_31_N_3_O_11_S: C 50.61%, H 5.49%, N 7.38%, S 5.63%. Found: C 50.57%, H 5.49%, N 7.32%, S 5.63%.

#### 4.1.4 4-(2′,3′,4′,6′-Tetra-*O*-acetyl-β-d-glucopyranosyloxy)-2- hydroxybenzaldehyde-3-thiosemicarbazone (**H**_**2**_ **L2**)

**(1)** (0.200g, 0.427 mmol, 1 equiv) was dissolved at reflux in ethanol (3 mL), then thiosemicarbazide (0.039 g, 0.427 mmol, 1 equiv) and few drops of CH_3_COOH were added. The reaction mixture was left stirring at reflux for 4 h. After that time, water was added and the reaction was cooled to 0 °C, filtered and washed with cold EtOH, obtaining **H**_**2**_ **L2** as a white solid (Y= 86%). IR (ATR, cm^–1^): ν­(OH) = 3277; ν­(NH) = 3291, 3180; ν­(C=O) = 1744; ν­(C=N) = 1596; ν­(C=S) = 1213, 1033. ^1^H NMR (500 MHz, DMSO-d6) δ 11.30 (s, 1H), 10.08 (s, 1H), 8.27 (1H), 8.06 (s, 1H), 7.88 (m, 2H), 6.47 (m, 2H), 5.57 (d, *J* = 7.9 Hz, 1H), 5.44 (t, *J* = 9.6 Hz, 1H), 5.02 (m, 2H), 4.22 (m, 2H), 4.05 (m, 1H), 2.02 (s, 6H), 2.01 (s, 3H), 1.97 (s, 3H). ^13^C NMR (126 MHz, DMSO-d_6_) δ 177.5, 170.7, 170.2, 169.5, 169.3, 160.0, 159.2, 147.8, 135.4, 133.0, 112.2, 109.5, 104.3, 98.12, 72.6, 72.4, 72.3, 70.9, 68.2, 61.9, 20.7, 20.6. HRMS: [M+Na]^+^ = 564.1248. Anal. Calcd for C_22_H_27_N_3_O_11_S · 0.5 H_2_O: C 47.99%, H 5.13%, N 7.63%, S 5.82%. Found: C 47.96%, H 5.03%, N 7.63%, S 5.82%.

#### 4.1.5 4-(2′,3′,4′,6′-Tetra-hydroxy-β-d-glucopyranosyloxy)-2-hydroxybenzaldehyde-4-ethyl-3- thiosemicarbazone (**H**_**2**_ **L3**)

**(2)** (200 mg; 0.666 mmol) was solubilized in hot methanol (8 mL) and solid 4-ethyl-3-thiosemicarbazide (79 mg; 0.666 mmol) was added, followed by three drops of glacial acetic acid. The resulting pale pink-yellow solution was refluxed for 4 h. The mixture was dried and the crude was recrystallized from absolute EtOH/Et_2_O. A white solid was obtained (*Y* = 43%). IR (ATR, cm^–1^): ν­(O–H) = 3304–3271; ν­(N–H) = 3163, 3003; ν­(C=N) = 1628; ν­(C=S) = 1072, 802. ^1^H NMR (400 MHz, DMSO-d_6_) δ 11.25 (s, 1H), 10.00 (s, 1H), 8.40 (t, *J* = 6.0 Hz, 1H), 8.28 (s, 1H), 7.87 (m, 1H), 6.56 (m, 2H), 5.31 (d, *J* = 5.2 Hz, 1H), 5.10 (d *J* = 4.8 Hz, 1H), 5.03 (d, *J* = 5.2 Hz, 1H), 4.83 (d, *J* = 7.6 Hz, 1H), 4.57 (t, *J* = 5.8 Hz, 1H), 3.68 (dd, *J* = 11.8, 5.2 Hz, 1H), 3.60–3.47 (m, 3H), 3.28–3.14 (m, 4H), 1.14 (t, *J* = 7.1 Hz, 3H). ^13^C NMR (101 MHz, DMSO-d6) δ 176.3, 159.8, 157.5, 139.1, 127.8, 114.6, 107.8, 103.4, 100.1, 77.1, 76.6, 73.2, 69.6, 60.6, 14.7. HRMS: [M + H]^+^= 402.1322. Anal. Calcd. for C_16_H_23_N_3_O_7_S · H_2_O: C 45.82%, H 6.01%, N 10.02%, S 7.64%. Found: C 45.60%, H 5.88%, N 10.06%, S 7.65%

#### 4.1.6 4-(2′,3′,4′,6′-Tetra-hydroxy-β-d-glucopyranosyloxy)-2- hydroxybenzaldehyde-3-thiosemicarbazone (**H**_**2**_ **L4**)

**(2)** (119 mg; 0.396 mmol) was solubilized in hot methanol (8 mL) and solid thiosemicarbazide (36 mg; 0.396 mmol) was added, followed by one drop of glacial acetic acid. The resulting solution was refluxed overnight, then it was cooled to r.t., reduced to half volume and Et_2_O (20 mL) was added. A white precipitate formed, which was filtered, washed with Et_2_O and dried *in vacuo*. A white solid was obtained (*Y* = 78%). IR (ATR, cm^–1^): ν­(OH + NH) = 3340–3160; ν­(C=N) = 1594; ν­(C=S) = 1065, 807. ^1^H NMR (400 MHz, DMSO-d_6_) δ 11.26 (b, 1H), 9.96 (s, 1H), 8.27 (s, 1H), 8.01 (b, 1H), 7.83 (m, 2H), 6.52 (m, *2* H), 5.30 (d, *J* = 5.2 Hz, 1H), 5.08 (d, *J* = 4.8 Hz, 1H), 5.02 (d, *J* = 5.2 Hz, 1H), 4.84 (d, *J* = 7.6 Hz, 1H), 4.56 (t, *J* = 5.8 Hz, 1H), 3.69 (m, 1H), 3.49 (m, 1H), 3.24–3.14 (m, 3H).

^13^C NMR (101 MHz, DMSO-d_6_) δ 175.57 (C=S), 160.30, 156.56, 139.92, 127.18, 116.03, 108.30, 103.87, 100.55, 77.56, 77.02, 73.63, 70.00, 61.01. HRMS: [M + H]^+^= 374.1016. Anal. Calcd. for C_14_H_19_N_3_O_7_S·2 H_2_O: C 41.07%, H 5.66%, N 10.26%, S 7.83. Found: C 40.98%, H 5.53%, N 10.06%, S 7.91%.

#### 4.1.7 [Cu­(HL1)­Cl] (**C1**)

**H**_**2**_ **L1** (67 mg, 0.12 mmol, 1 equiv) was dissolved in 2 mL of THF, then 2 drops of NaOH 1 M were added, followed by a solution of CuCl_2_·2H_2_O (20 mg, 0.12 mmol, 1 equiv) in 2 mL of THF. The reaction was left stirring at r.t. under N_2_ for 3 h, then 15 mL of Et_2_O were added. The green solid was then recovered by filtration and washed with Et_2_O (*Y* = 67%). IR (ATR, cm^–1^): ν­(N–H) = 3228, 2980; ν­(C=O) = 1752; ν­(C = N) = 1530; ν­(C=S) = 1045. HRMS: [M + Na]^+^= 689.0469. Anal. Calcd. for C_24_H_30_ClCuN_3_O_11_S · 2 H_2_O: C 40.97%, H 4.87%, N 5.97%, S 4.56%. Found: C 40.93%, H 4.49%, N 6.05%, S 4.61%.

#### 4.1.8 [Cu­(HL2)­Cl] (**C2**)

**H**_**2**_ **L2** (100 mg, 0.18 mmol, 1 equiv) was dissolved in 2 mL of methanol and 1 mL of THF, then 2 drops of NaOH 1 M were added, followed by a solution of CuCl_2_ · 2H_2_O (31.5 mg, 0.18 mmol, 1 equiv) in 2 mL of methanol. The reaction was left stirring at r.t. under N_2_ for 3 h, then 15 mL of Et_2_O are added. The green solid was then recovered by centrifugation and washed with Et_2_O (*Y* = 58%). IR (ATR, cm^–1^): ν­(N–H) = 3170; ν­(C=O) = 1743; ν­(C=N) = 1606; ν­(C=S) = 1033. HRMS: [M+Na]^+^ = 661.0163. Anal. Calcd. for C_22_H_26_ClCuN_3_O_11_S·2.5 H_2_O: C 38.60%, H 4.56%, N 6.14%, S 4.68%. Found: C 38.27%, H 4.14%, N 6.11%, S 4.71%.

#### 4.1.9 [Cu­(HL3)­Cl] (**C3**)

**H**_**2**_ **L3** (88 mg, 0.22 mmol, 1 equiv) was dissolved in 2 mL of methanol, then 2 drops of NaOH 1 M were added, followed by a solution of CuCl_2_·2H_2_O (38 mg, 0.22 mmol, 1 equiv) in 3 mL of MeOH. The reaction was left stirring at r.t. under N_2_ for 3 h, then the dark green/blue solid was filtered and washed with methanol (*Y* = 61%). IR (ATR, cm^–1^): ν­(N–H) = 3206; ν­(C–H) = 2981; ν­(C=N) = 1601; ν­(C=S) = 1019. HRMS: [M+Na]^+^= 521.0049. Anal. Calcd. for C_16_H_22_ClCuN_3_O_7_S·2 H_2_O: C 35.89%, H 4.89%, N 7.85%. Found: C 35.75%, H 4.89%, N 7.88%, S 7.94%.

#### 4.1.10 [Cu­(HL4)­Cl] (**C4**)

**H**_**2**_ **L4** (115 mg, 0.31 mmol, 1 equiv) was dissolved in 2 mL of methanol, then 3 drops of NaOH 1 M were added, followed by a solution of CuCl_2_·2H_2_O (52.5 mg, 0.31 mmol, 1 equiv) in 3 mL of methanol. The reaction was left stirring at r.t., under N_2_ for 3 h, then the dark green/blue solid was filtered and washed with methanol (*Y* = 66%). IR (ATR, cm^–1^): ν­(OH) = 3294; ν­(NH) = 3198, 3069; ν­(C=N) = 1608; νC=S = 1039. HRMS: [M+Na]^+^ = 492.9736. Anal. Calcd. for C_14_H_18_ClCuN_3_O_7_S·2.5 H_2_O: C 32.56%, H 4.49%, N 8.14%, S 6.21%. Found: C 32.83, H 4.40, N 7.67, S 6.33%.

### 4.2 Experiments with Cultured Human Cancer Cells

The compounds to analyze were prepared in DMSO just prior to treatment, and the resulting solution was diluted into the culture medium to achieve 0.5% DMSO, a level that showed no impact on cell viability. Cisplatin was dissolved in 0.9% sodium chloride solution. Sulphorhodamine-B (SRB) and cisplatin were obtained from Sigma Chemical Co, St. Louis, MO, USA.

### 4.3 Cell Cultures

Human colon (HCT-15), breast (MCF-7) and pancreatic (PSN-1) carcinoma cell lines were obtained by American Type Culture Collection (ATCC, Rockville, MD, USA). Human thyroid (B-CPAP) carcinoma cells were obtained by Leibniz Institute (DSMZ-German Collection of Microorganisms and Cell Cultures GmbH, Braunschweig, Germany). Human ovarian 2008 cancer cells and their cisplatin-resistant subline, C13* cells, were kindly provided by Prof. G. Marverti (Dept. of Biomedical Science of Modena University, Italy). All cell lines were sustained in exponential growth conditions at 37 °C in an environment containing 5% carbon dioxide by using RPMI-1640 (HCT-15, MCF-7, PSN-1, B-CPAP, 2008 and C13*) medium (Euroclone) supplemented by the addition of 10% fetal calf serum (EuroClone, Milan, Italy), antibiotics (50 units/mL penicillin and 50 μg/mL streptomycin) and 2 mM l-glutamine.

### 4.4 Cytotoxicity Assay

The growth inhibitory effect toward tumor cells was evaluated by means of sulphorhodamine-B assay. Briefly, (3–8) × 10^3^ cells/well (depending on cell type) were seeded in 96-well microplates in growth medium (100 μL). After 24 h, the medium was removed and replaced with a fresh one containing the compound to be studied at the appropriate concentration. Triplicate cultures were established for each treatment. After 72 h, the experiment was terminated by carefully overlaying the cells with a chilled 30% trichloroacetic acid (TCA) solution (50 μL), followed by fixation at 4 °C for 1 h. The plates were then stained with 50 μL of 0.4% sulforhodamine B (SRB) for 20 min. Excess dye was removed, and the protein-bound SRB was solubilized by adding 100 μL of 10 mM Tris buffer (pH = 10.5) to each well. Absorbance was measured at 540 nm, using 690 nm as the reference wavelength by using a Bio-Rad 680 microplate reader. Mean absorbance for each drug dose was expressed as a percentage of the control untreated well absorbance and plotted vs drug concentration. IC_50_ values, the drug concentrations that reduce the mean absorbance to 50% of those in the untreated control wells, were calculated by the four-parameter logistic (4-PL) model. Evaluation was based on means from at least three independent experiments.

### 4.5 Spheroid Cultures and Acid Phosphatase (APH) Assay

Spheroids were generated by plating 2.5 × 10^3^ PSN-1 and 2008 cells per well into round-bottom, nontreated 96-well plates (Greiner Bio-One, Kremsmünster, Austria) using phenol-red-free RPMI medium (Sigma Chemical Co., St. Louis, MO, USA) supplemented with 10% fetal calf serum and 20% methylcellulose stock solution. Cell viability within the 3D spheroids was assessed using a modified APH assay.^31^ IC_50_ values—defined as the drug concentration that lowers the absorbance at 405 nm to 50% of that observed in untreated controls—were determined using a four-parameter logistic (4-PL) model.

### 4.6 GLUT Expression

Expression levels in cancer cells were evaluated by means of the GLUT1 Colorimetric Cell-Based ELISA kit (Boster Biological Technology, Pleasanton CA, USA). Approximately 2 × 10^4^ cells were seeded in 96-well microplates, and GLUT1 expression was detected at 450 nm, using a BioRad 680 microplate reader (BioRad Laboratories S.r.L.), following the manufacturer’s instructions.

### 4.7 Cellular Copper Uptake

The cellular copper uptake was evaluated by using concentrations and time exposures that did not affect cell viability. PSN-1 cancer cells (2.5 × 10^6^) were seeded in 75 cm^2^ flasks in growth medium (20 mL). After 24h the medium had been replaced, and the cells were incubated with tested compounds for 24 or 36 h. Subsequently, cell monolayers were washed twice with cold PBS (2 mL) and harvested. Samples were subjected to three freeze/thaw cycles at −80 °C and then vigorously vortexed. Aliquots were removed for the determination of protein content by the Bradford protein assay (BioRad). The samples were treated with 1 mL of highly pure nitric acid (Cu: < 0.005 mgkg^–1^, TraceSELECT Ultra, Sigma Chemical Co.) and transferred into a Teflon microwave vessel. Samples were then submitted to a standard procedure using a speed wave MWS-3 Berghof instrument (Eningen, Germany). After cooling, each mineralized sample was analyzed for iron using a Varian AA Duo graphite furnace atomic absorption spectrometer (GF-AAS, Varian, Palo Alto, CA, USA) at 324 nm. The calibration curve was obtained using known concentrations of standard solutions purchased from Sigma Chemical Co. The results were expressed as ng of Cu per mg of protein.

### 4.8 Protein Disulfide Isomerase (PDI) Activity

PDI reductase activity was evaluated by monitoring the PDI-mediated reduction of insulin in the presence of increasing concentrations of the test compounds, using the PROTEOSTAT PDI assay kit (Enzo Life Sciences, Lausen, Switzerland). All procedures were carried out following the manufacturer’s protocol and as detailed in previous reports.^30,33^

### 4.9 Confocal Microscopy Morphological Analyses

PSN-1 cells were seeded into 8-well tissue-culture slides (BD Falcon, Bedford, MA, USA) at 5 × 10^4^ cells/well (0.8 cm^2^). After 24 h, the cells were washed twice with PBS, and following 24 h of treatment with IC_50_ doses of the tested compound, cells were stained for 5 min with 10 μg/mL of Hoechst 33342 (Sigma-Aldrich, St. Louis, MI, USA) in PBS. Samples were examined at 10 × magnification in a Zeiss LSM 800 confocal microscope using the Zeiss ZEN 2.3 software system.

### 4.10 Transmission Electron Microscopy (TEM) Analyses

Approximately 1 × 10^6^ PSN-1 cells were seeded into 24-well plates and allowed to grow for 24 h before being exposed to the test compounds at their respective IC_50_ values for an additional 24 h. Following treatment, the cells were rinsed with cold PBS, collected, and immediately fixed in 1.5% glutaraldehyde prepared in 0.2 M sodium cacodylate buffer (pH 7.4). After buffer rinsing and postfixation with 1% OsO_4_ in the same cacodylate buffer, samples were dehydrated and embedded in Epon–Araldite resin. Semithin sagittal sections (1 μm) were stained with toluidine blue, while ultrathin sections (≈90 nm) were contrasted using uranyl acetate and lead citrate. Electron micrographs were obtained using a Hitachi H-600 transmission electron microscope (Tokyo, Japan) operated at 75 kV, and images were formatted using Corel Draw 11.

### 4.11 Statistical Analysis

All data are presented as the mean ± SD from at least three independent measurements derived from separate cell cultures. Statistical differences among groups were assessed using ANOVA followed by the Tukey–Kramer post hoc test (*p < 0.05, **p < 0.01) with GraphPad software.

## Supplementary Material



## Data Availability

Research data underlying this work are available throughout the manuscript and Supporting files.

## Notes

ATR-IR, ^1^H- and ^13^C NMR spectra of **H_2_L1-H_2_L4** and **C1–C4**; ESI-MS spectra of **H_2_L1-H_2_L4** and **C1–C4**; UV–visible spectra over time of complexes **C1–C4** (PDF)
