# Supplementary material for: Glyco-Conjugation of Thiosemicarbazone Copper(II) Complexes Enhances Selectivity toward Cancer Cells Overexpressing GLUT1
Source: ACS Omega. 2026 May 29;11(22):32912–22. doi: 10.1021/acsomega.6c02319 (PMC13261448; doi:10.1021/acsomega.6c02319)

# *Glyco-conjugation of thiosemicarbazone copper(II) complexes enhance selectivity towards cancer cells overexpressing GLUT1*

Francesca Miglioli[a,c], Alessio Zavaroni [a,c], Cristina Marzano[b], Diego Montagner[c], Mauro Carcelli [a], Valentina Gandin[b,*], Dominga Rogolino[a,*]

[a] *Department of Chemistry, Life Sciences, Environmental Sustainability, University of Parma, Parco Area delle Scienze 11/A, 43124 Parma, Italy*

[b] *Department of Pharmaceutical and Pharmacological Sciences, University of Padova*

*via F. Marzolo 5, 35131 Padova – Italy*

[c] *Department of Chemistry, Maynooth University, Maynooth, Ireland*

**Corresponding authors contributed equally to the work*

**Figure S1.** $^{1}H$ NMR (top) and $^{13}C\{^{1}H\}$ NMR (bottom) for compound **(1)** dissolved in $CDCl_3$.

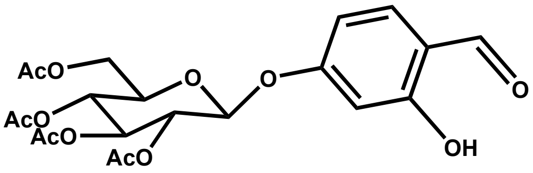


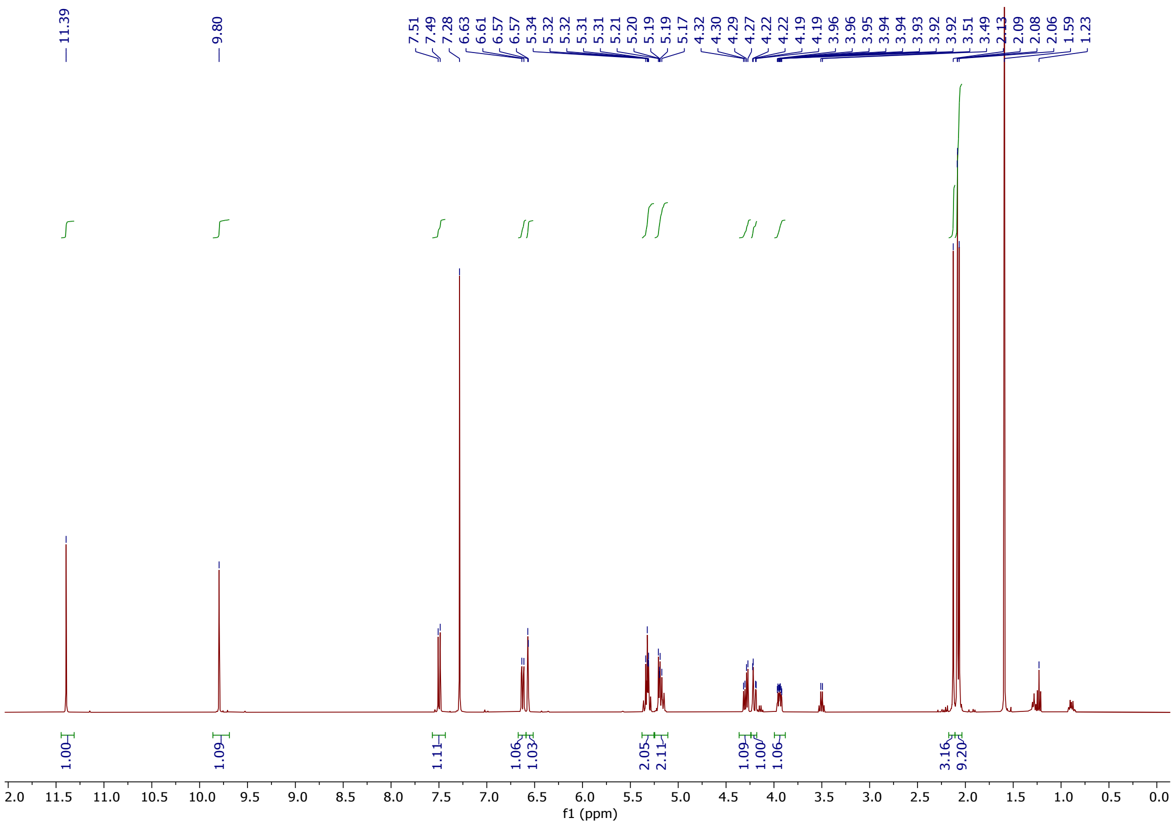


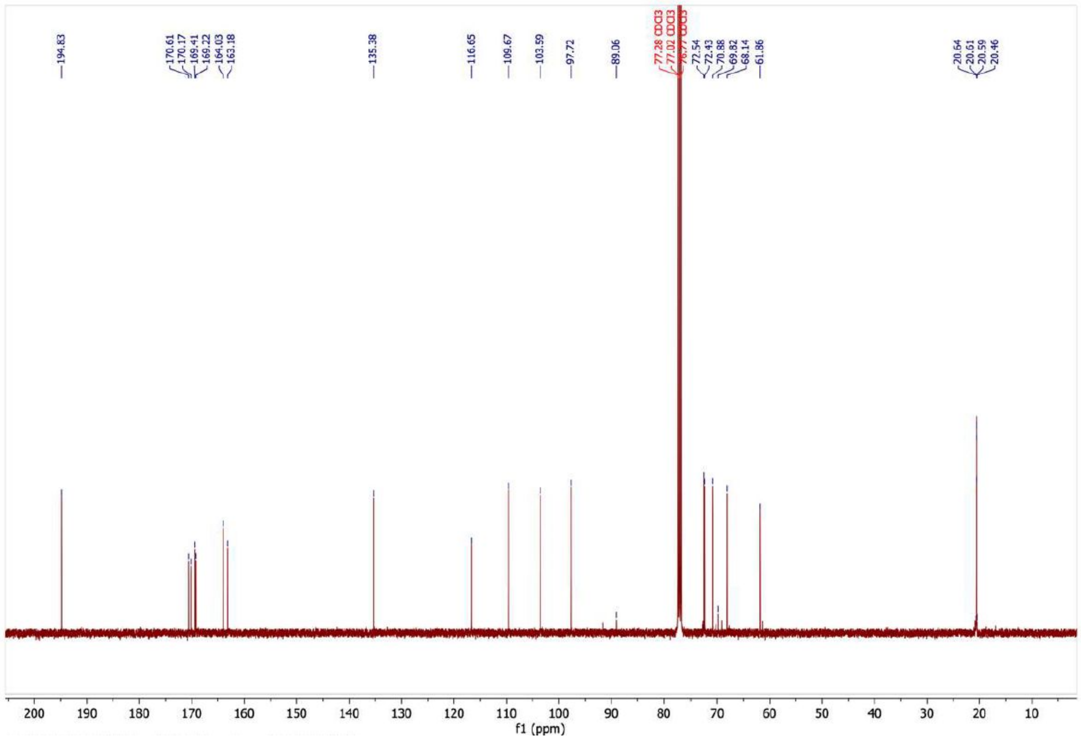

**Figure S2.** ATR-IR of compound **(1)**.

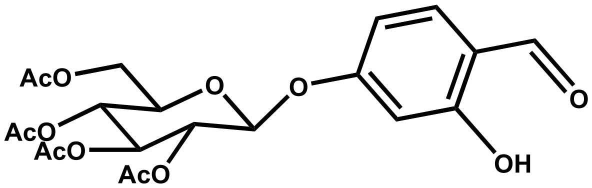


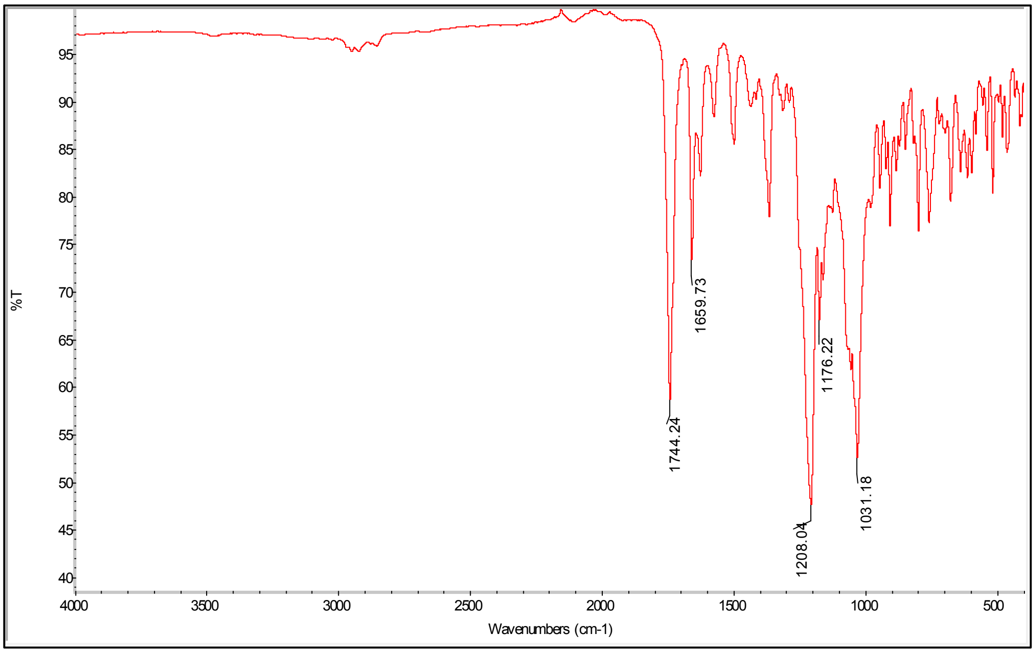


**Figure S3.** ESI-MS (positive ions) for compound **(1)** dissolved in methanol.

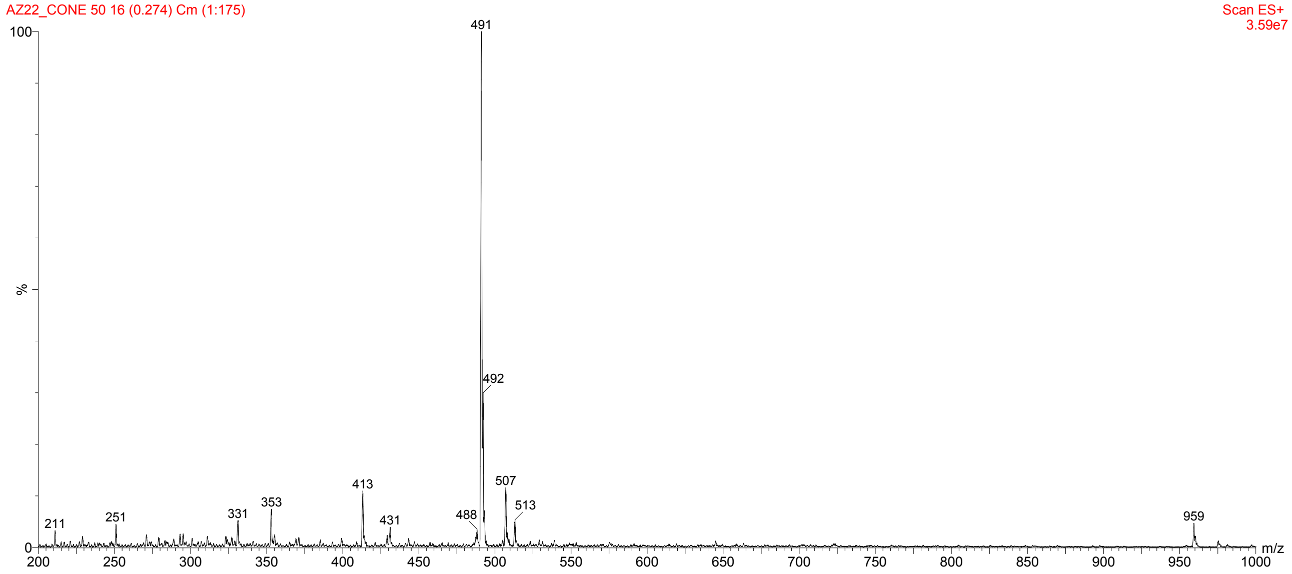

**Figure S4.** $^{1}$H NMR (top) and $^{13}$C{$^{1}$H} NMR (bottom) for compound **(2)** dissolved in DMSO-d$_6$.

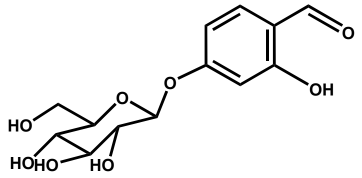


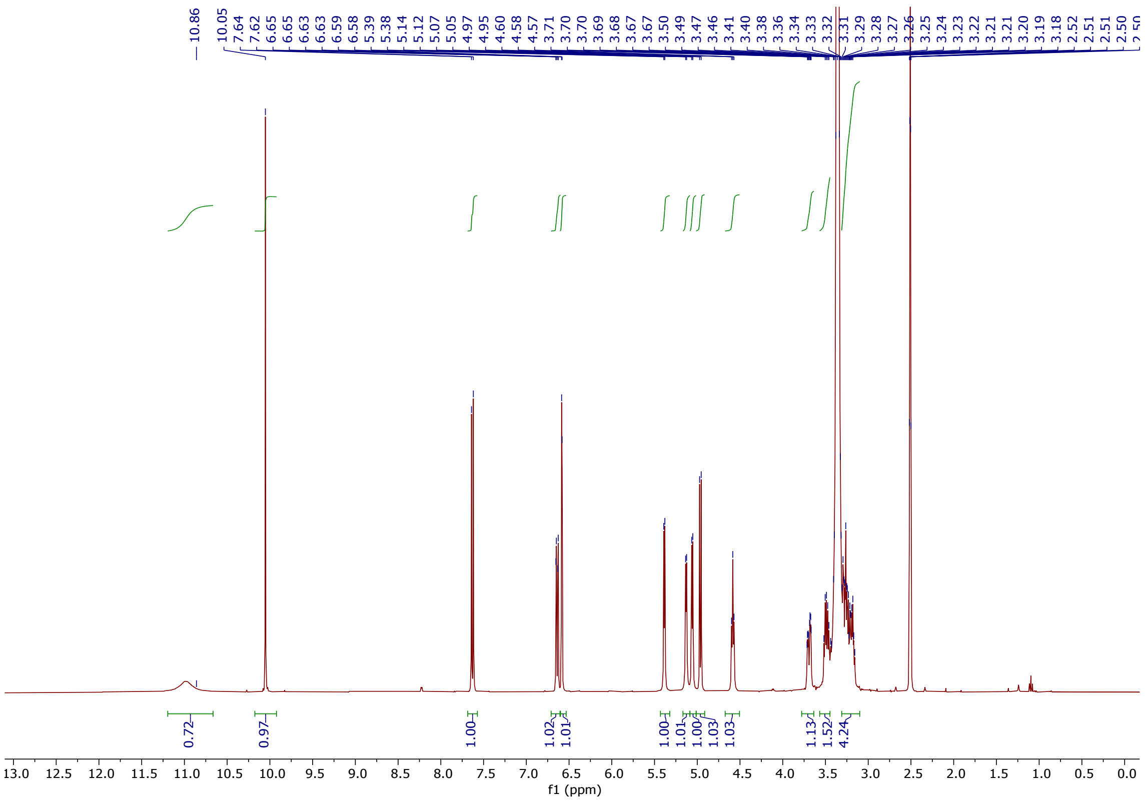


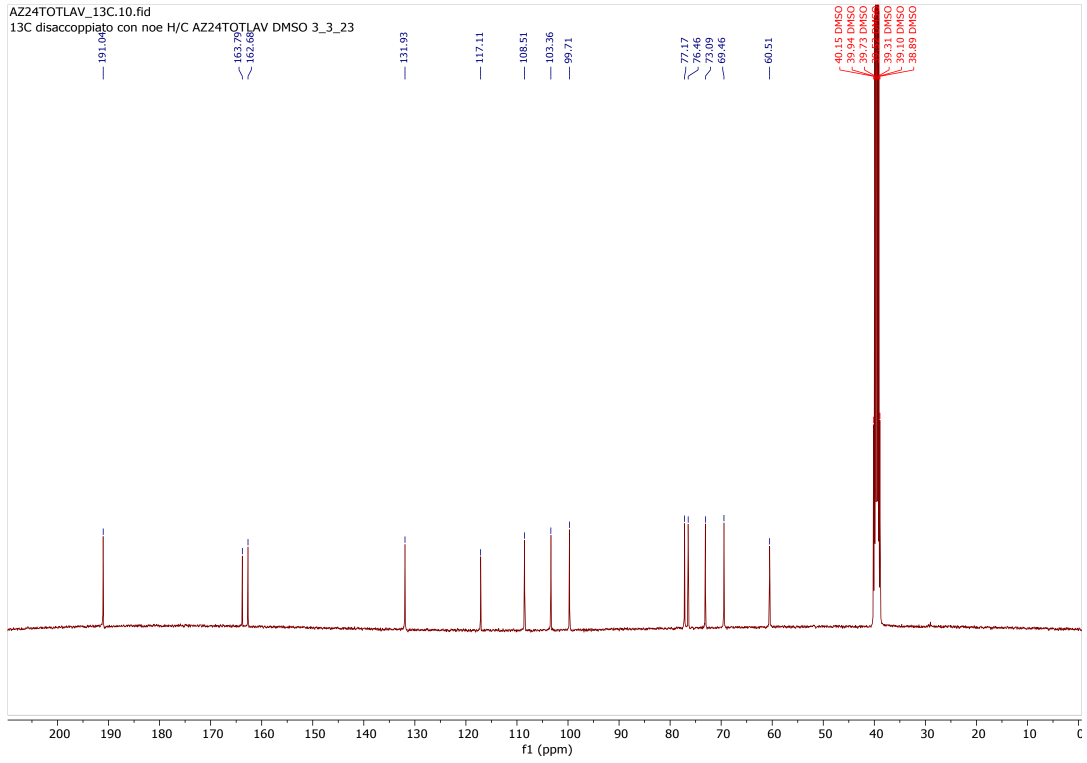

**Figure S5**. ATR-IR of compound **(2)**.

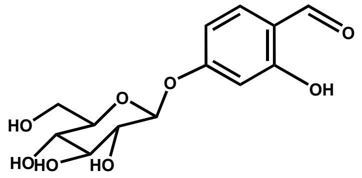


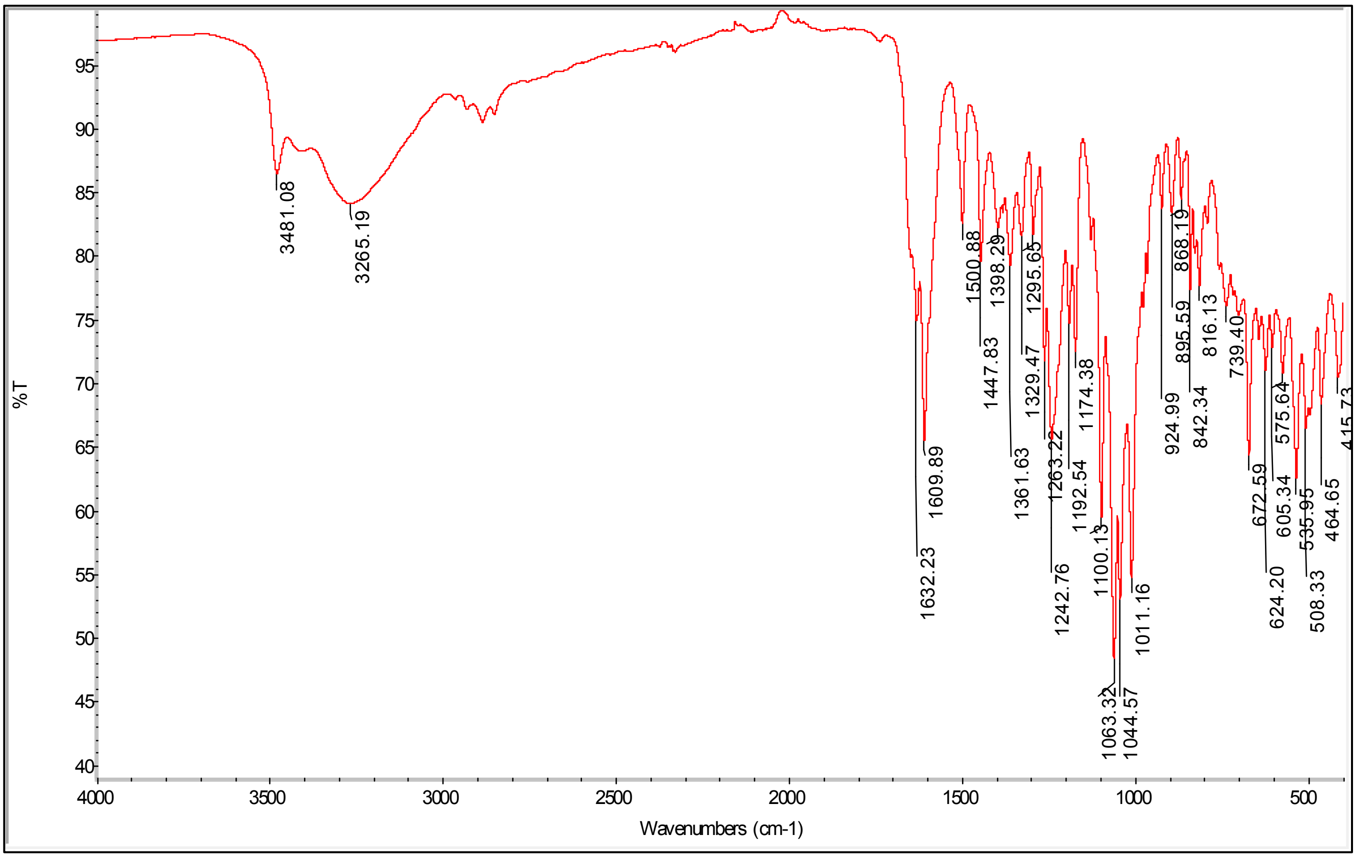


**Figure S6.** ESI-MS (positive ions) for compound **(2)** dissolved in methanol.

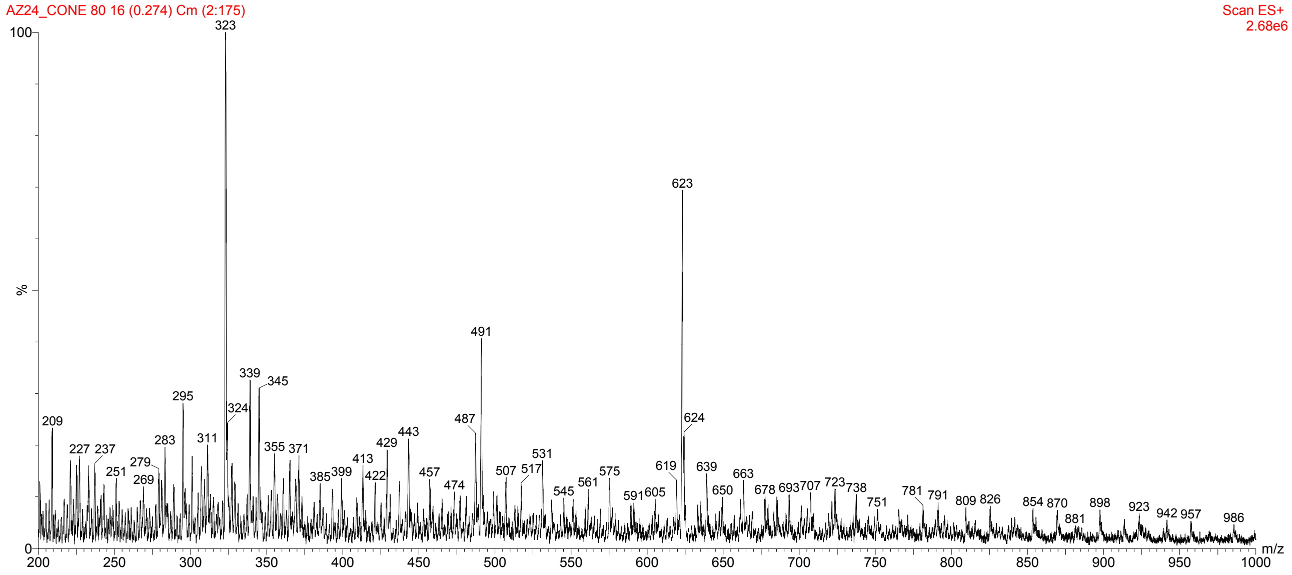

**Figure S7.** $^{1}H$ NMR (top) and $^{13}C$ NMR (bottom) for ligand $\mathbf{H_2L1}$ dissolved in DMSO-$d_6$.

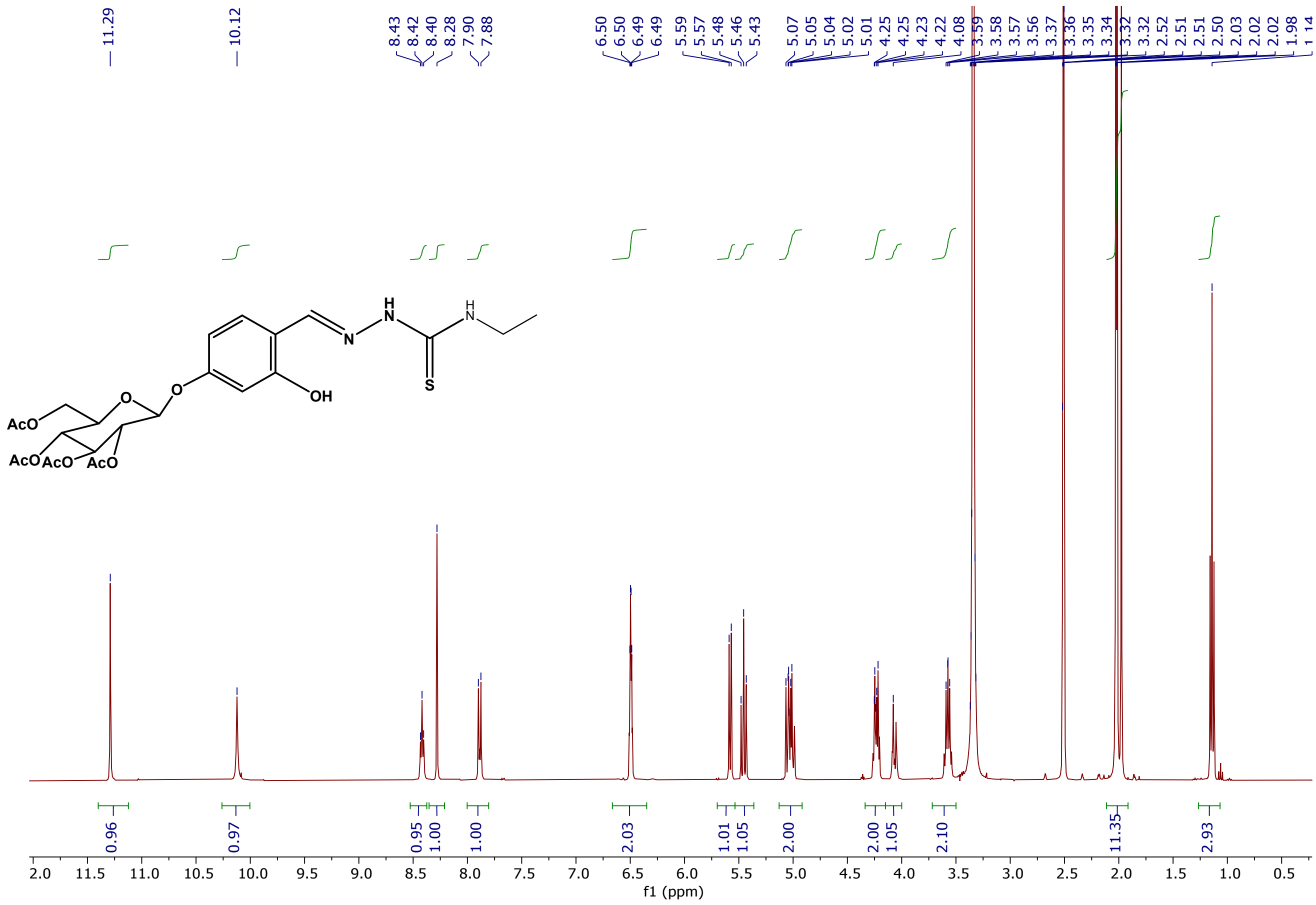


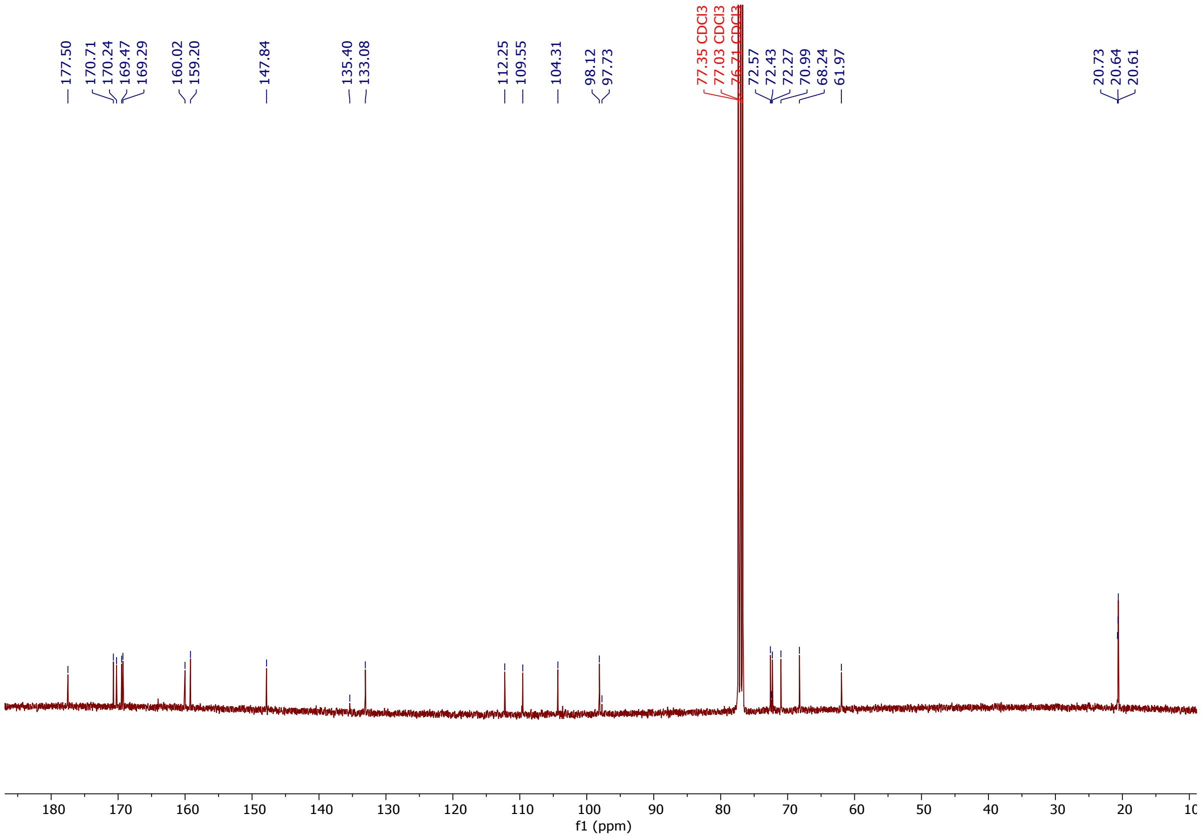

**Figure S8**. ATR-IR of compound **H$_2$L1**.

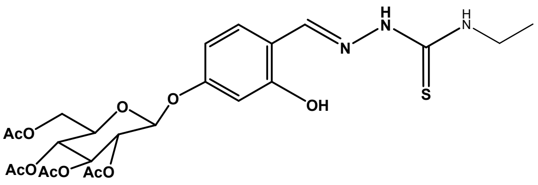

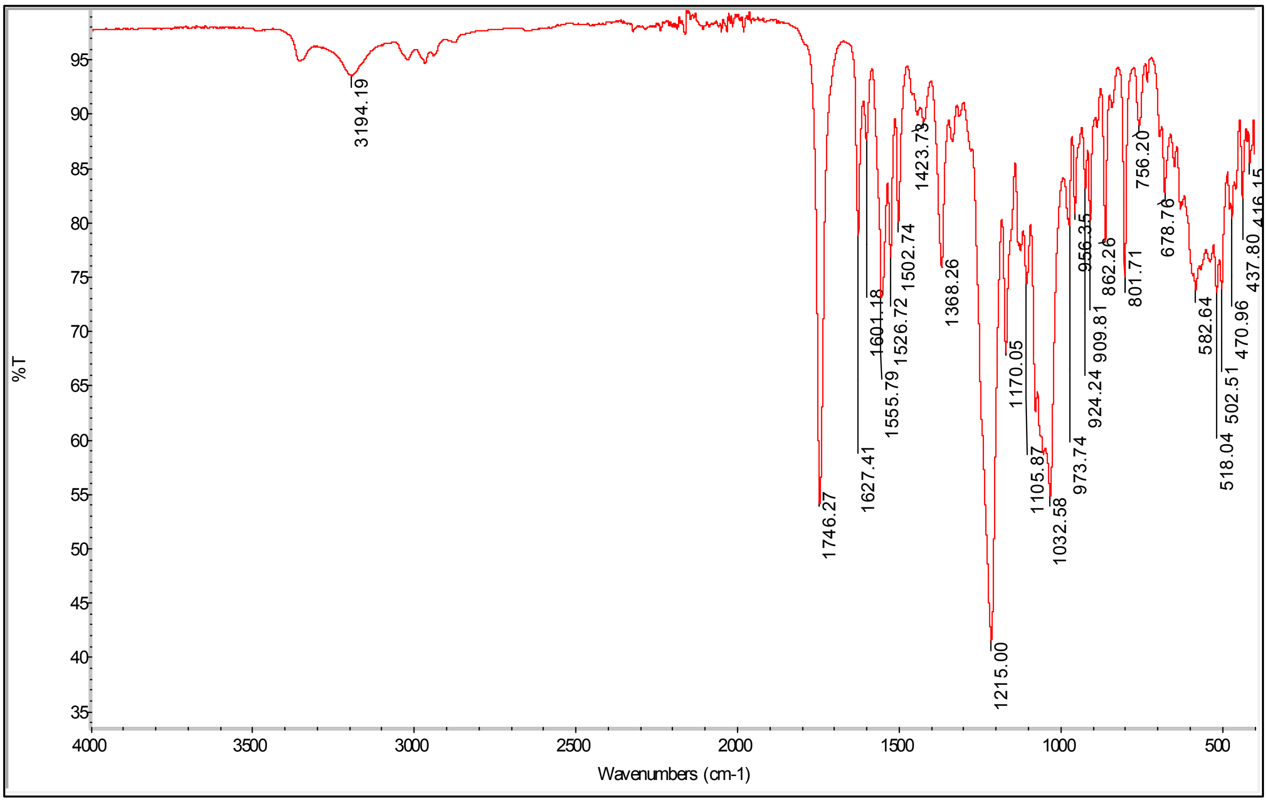


**Figure S9.** ESI-MS (positive ions) for compound **H$_2$L1**dissolved in methanol.

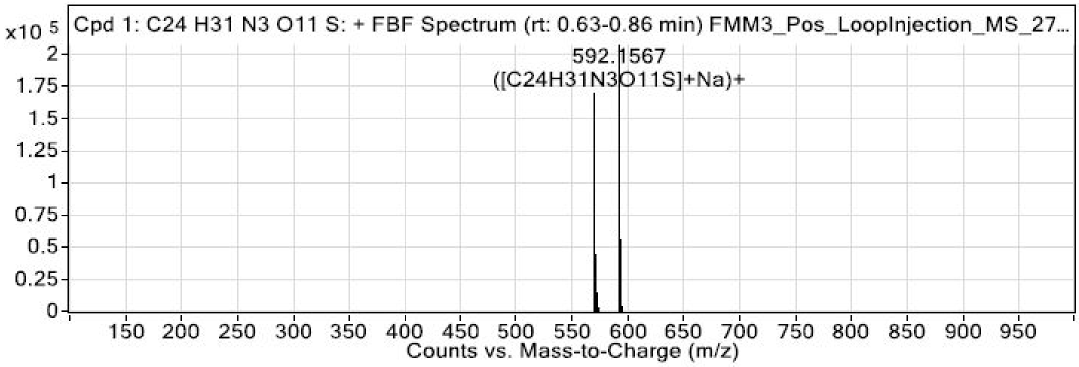

**Figure S10.** $^1H$ NMR (top) and $^{13}C$ NMR (bottom) for ligand **$H_2L2$** dissolved in DMSO-$d_6$.

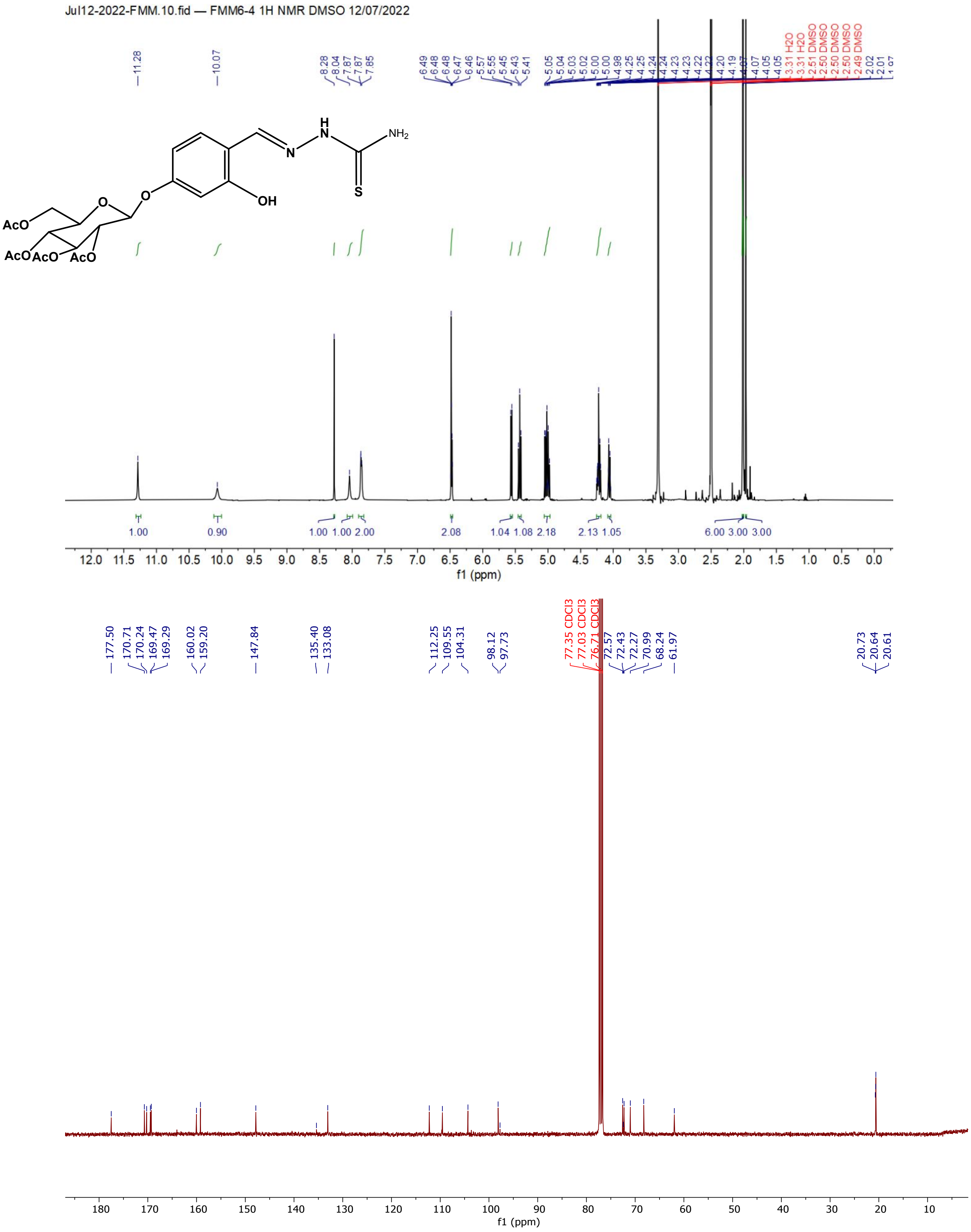

**Figure S11**. ATR-IR of compound **$H_2L2$**.

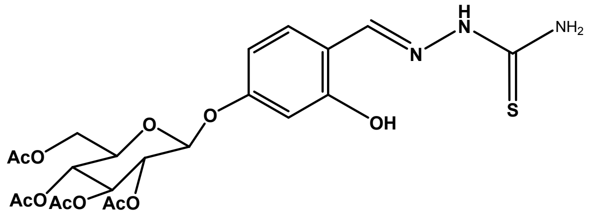


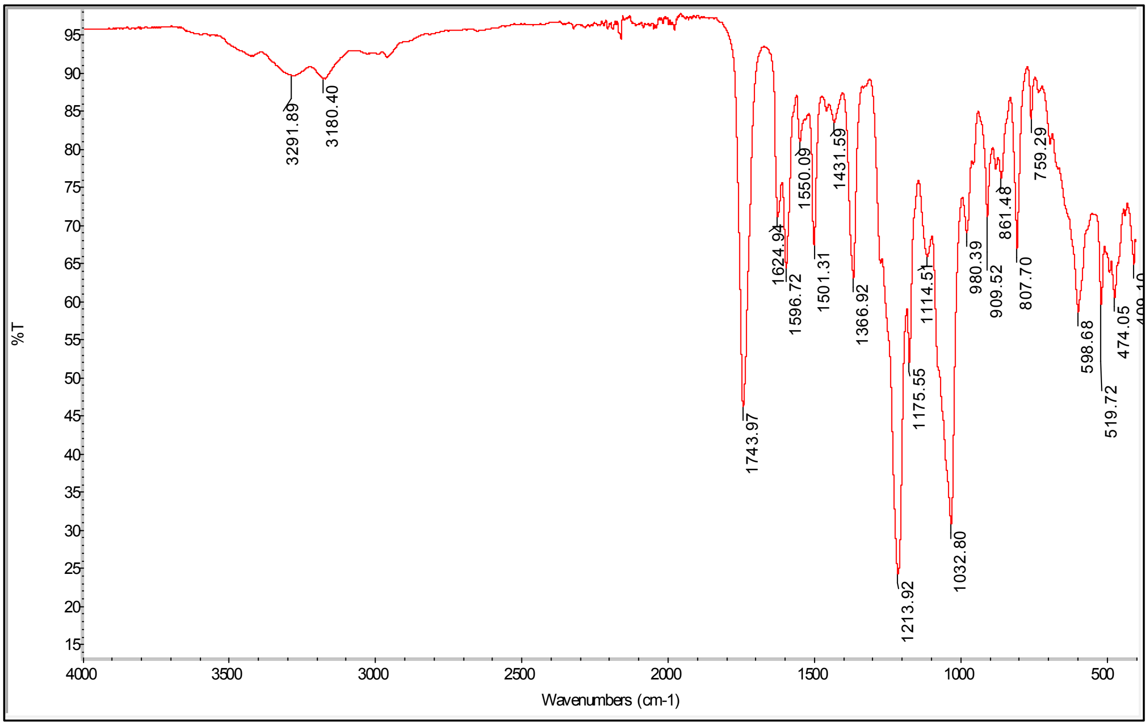


**Figure S12.** ESI-MS (positive ions) for compound **$H_2L2$** dissolved in methanol.

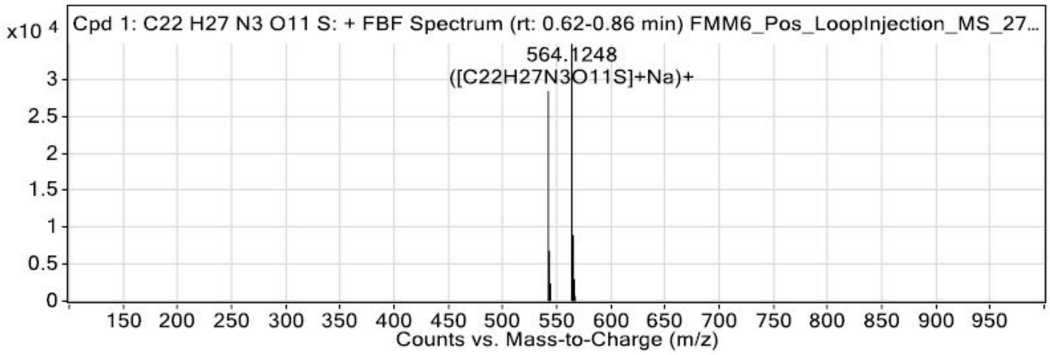

**Figure S13.** $^{1}H$ NMR (top) and $^{13}C$ NMR (bottom) for ligand **$H_2L3$** dissolved in DMSO-$d_6$.

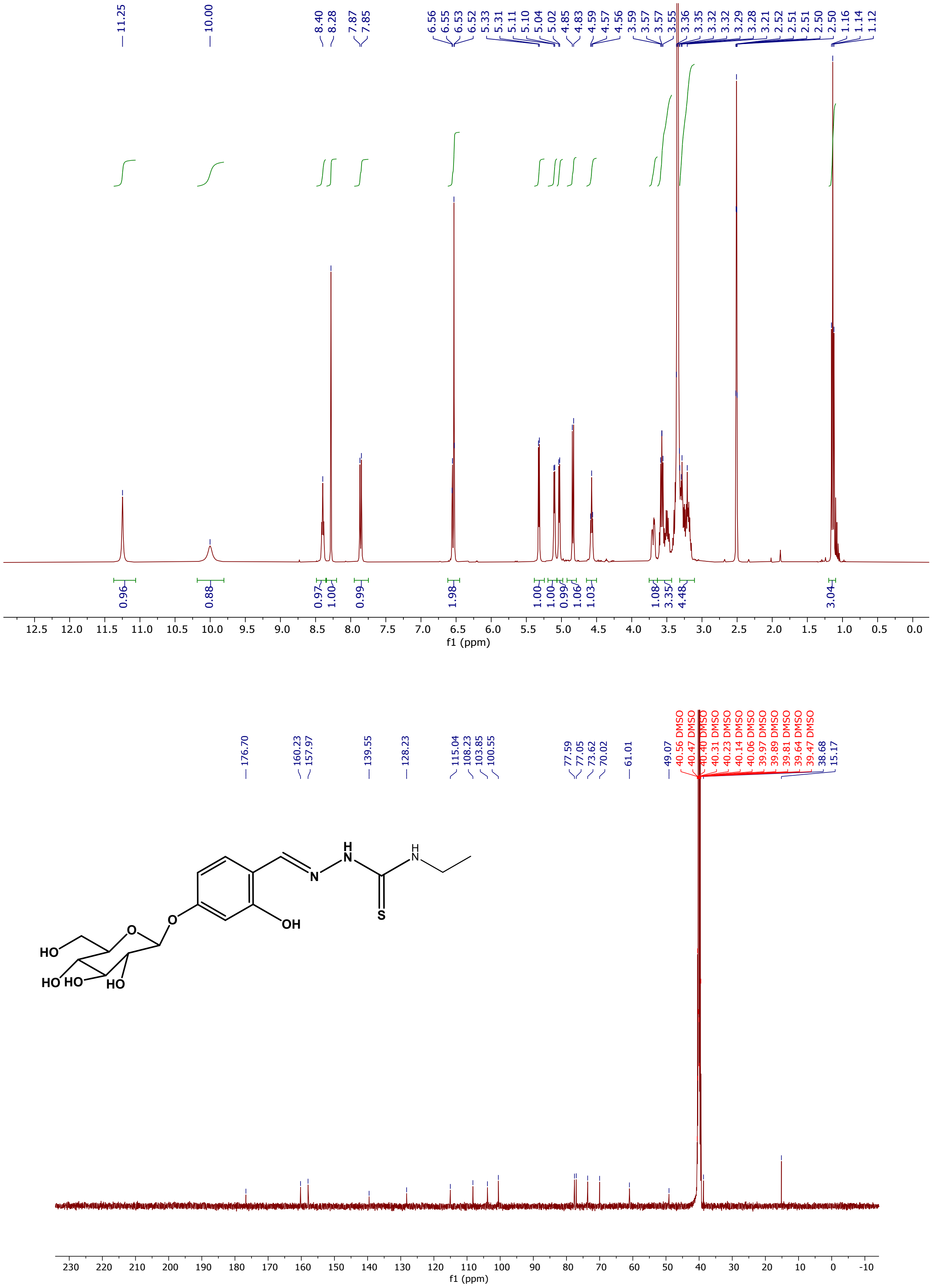

**Figure S14**. ATR-IR of compound $\mathbf{H_2L3}$.

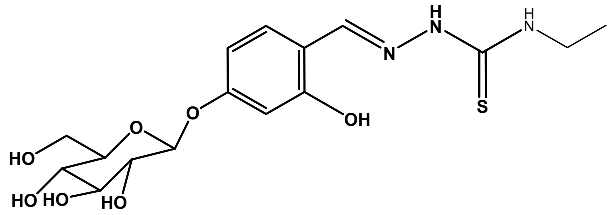


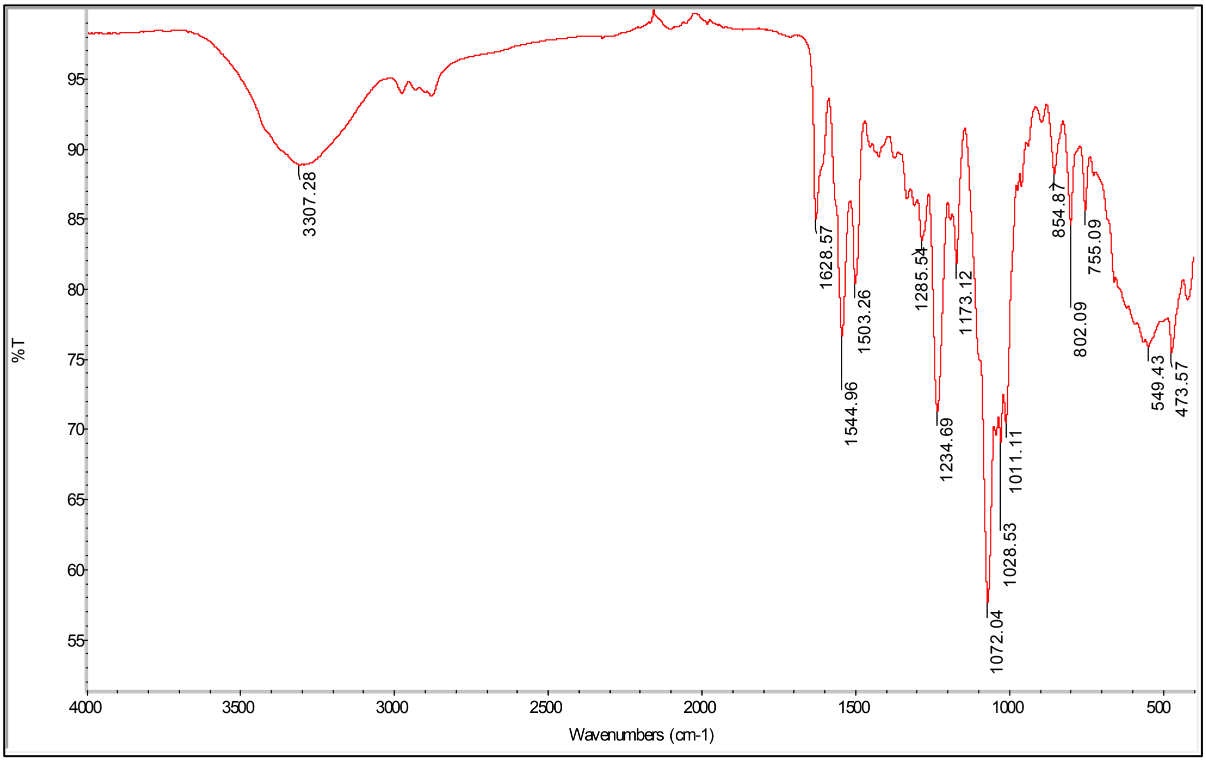


**Figure S15.** ESI-MS (positive ions) for compound $\mathbf{H_2L3}$ dissolved in methanol.

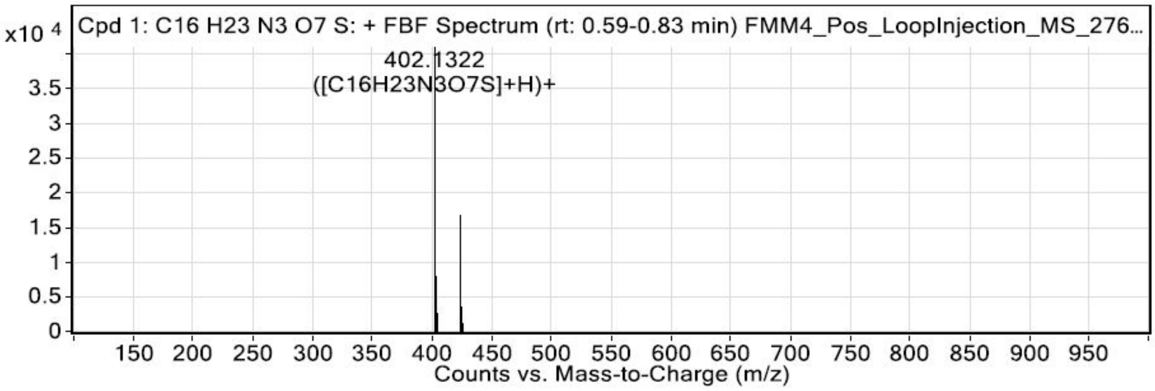

**Figure S16.** $^1$H NMR for ligand **$H_2$L4** dissolved in DMSO-$d_6$.

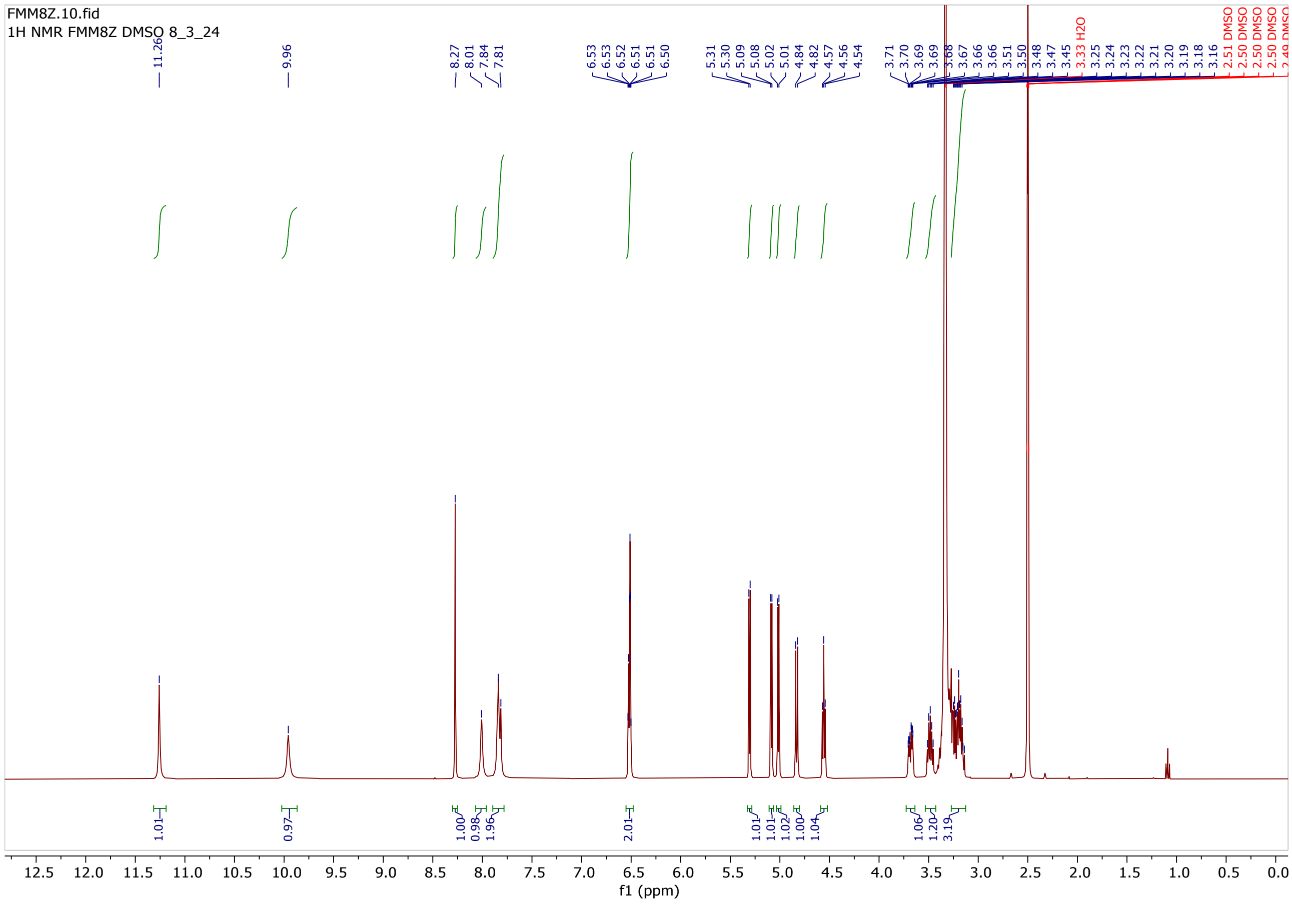


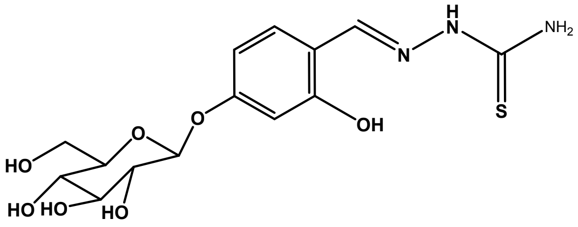

**Figure S17**. ATR-IR of compound **$H_2L4$**.

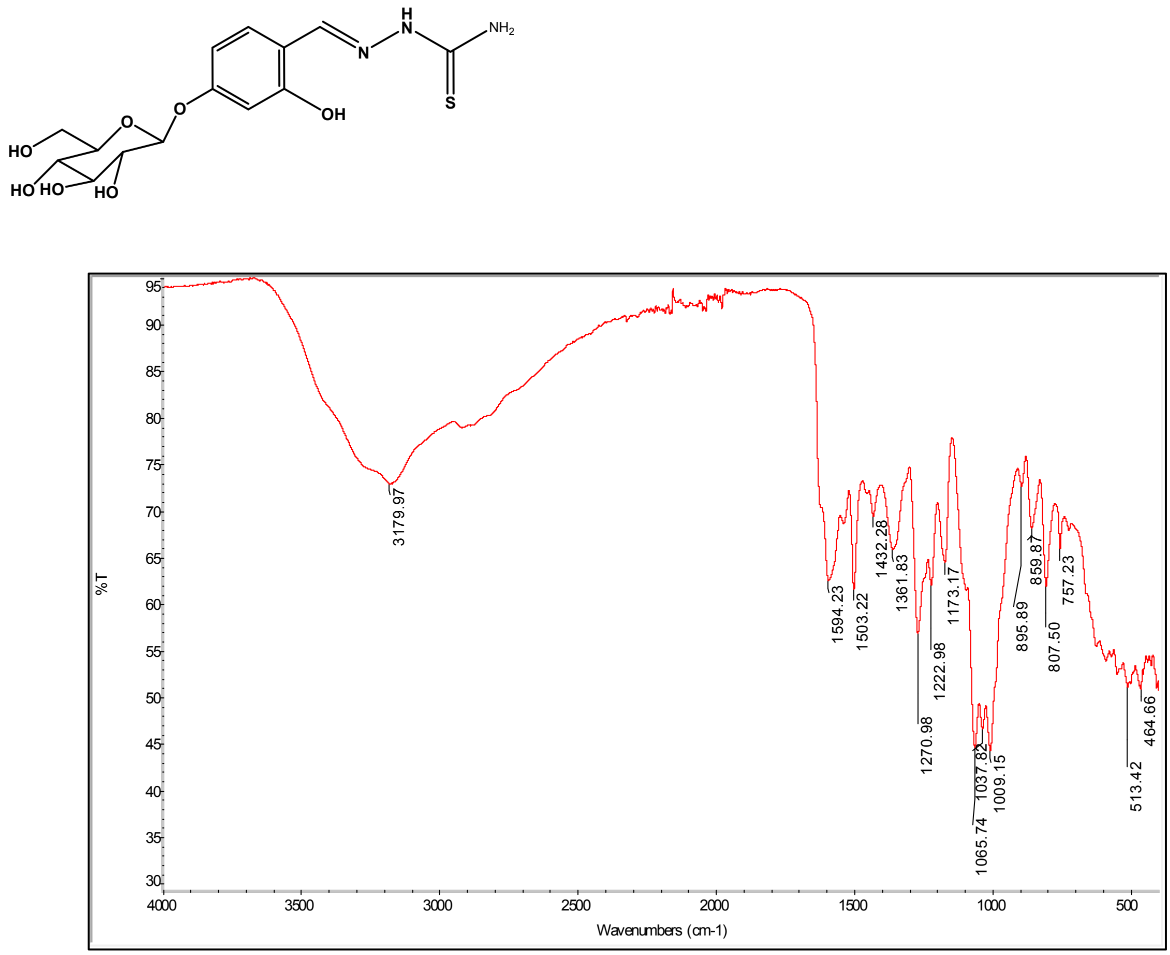


**Figure S18.** ESI-MS (positive ions) for compound **$H_2L4$** dissolved in methanol.

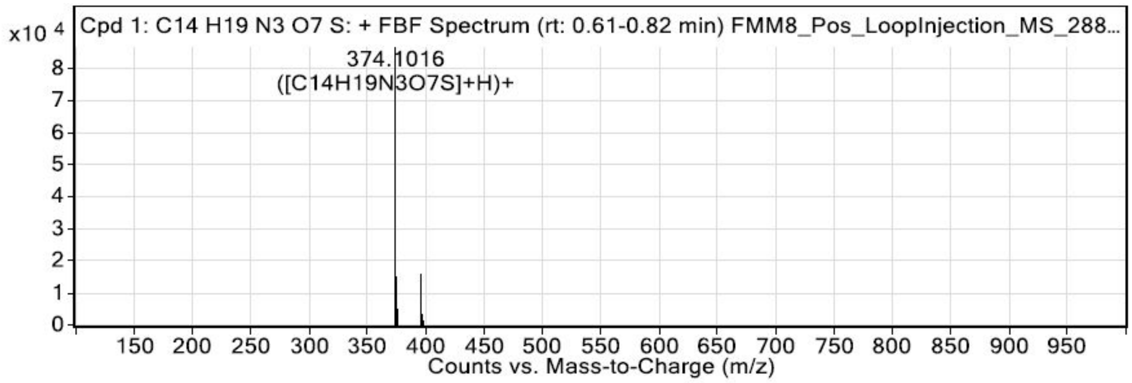

**Figure S19**. ATR-IR of compound **C1**.

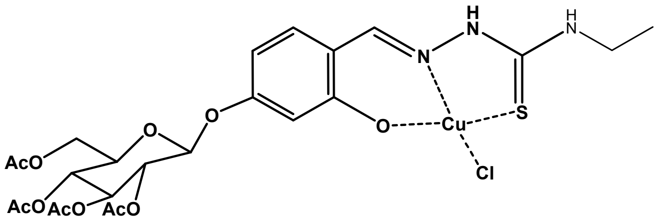


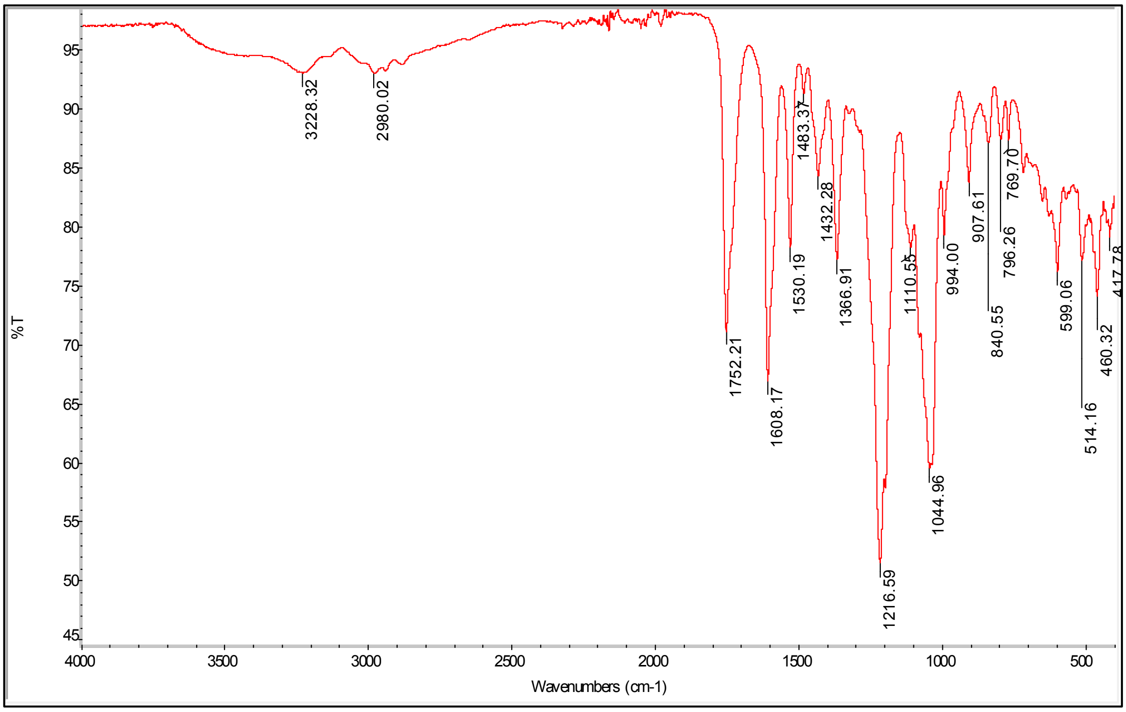


**Figure S20**. ESI-MS (positive ions) for compound **C1** dissolved in methanol.

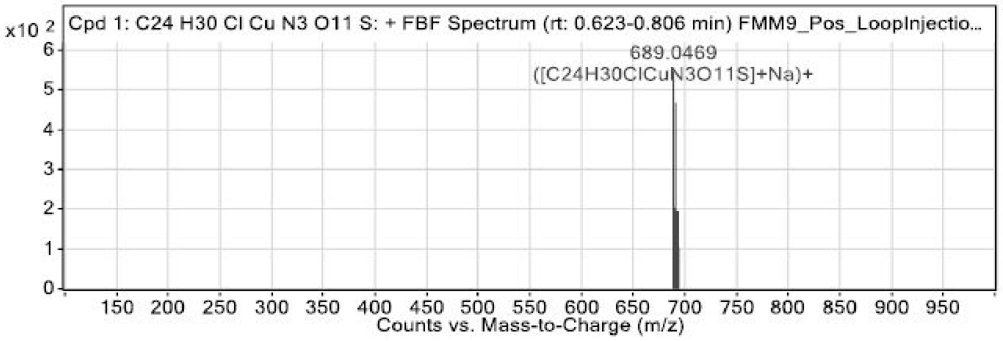

**Figure S21**. ATR-IR of compound **C2**.

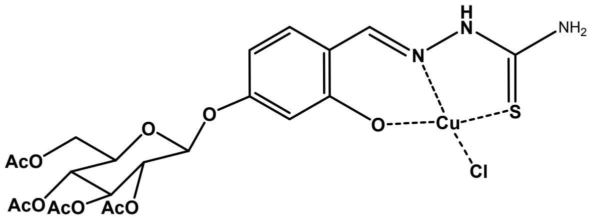


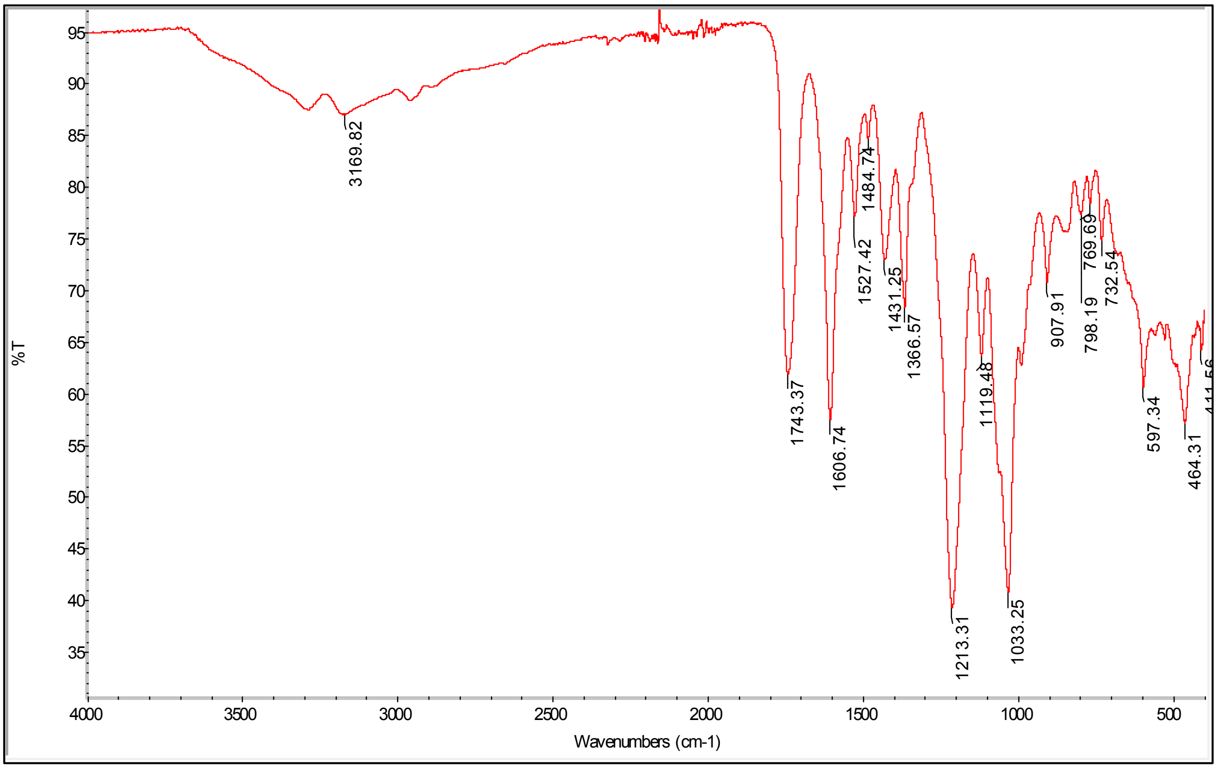


**Figure S22**. ESI-MS (positive ions) for compound **C2** dissolved in methanol.

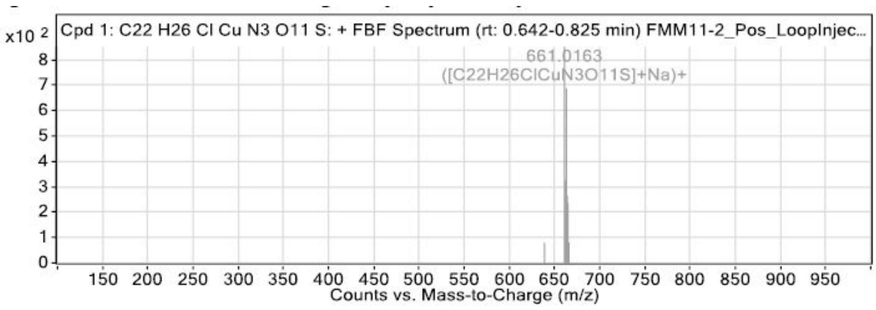

**Figure S23**. ATR-IR of compound **C3**.

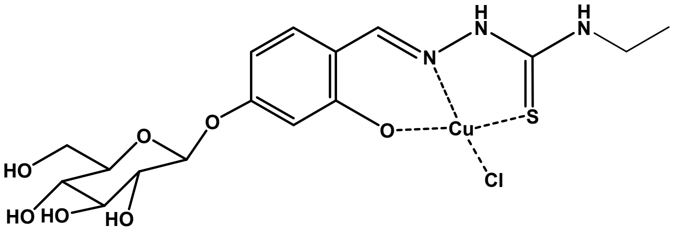


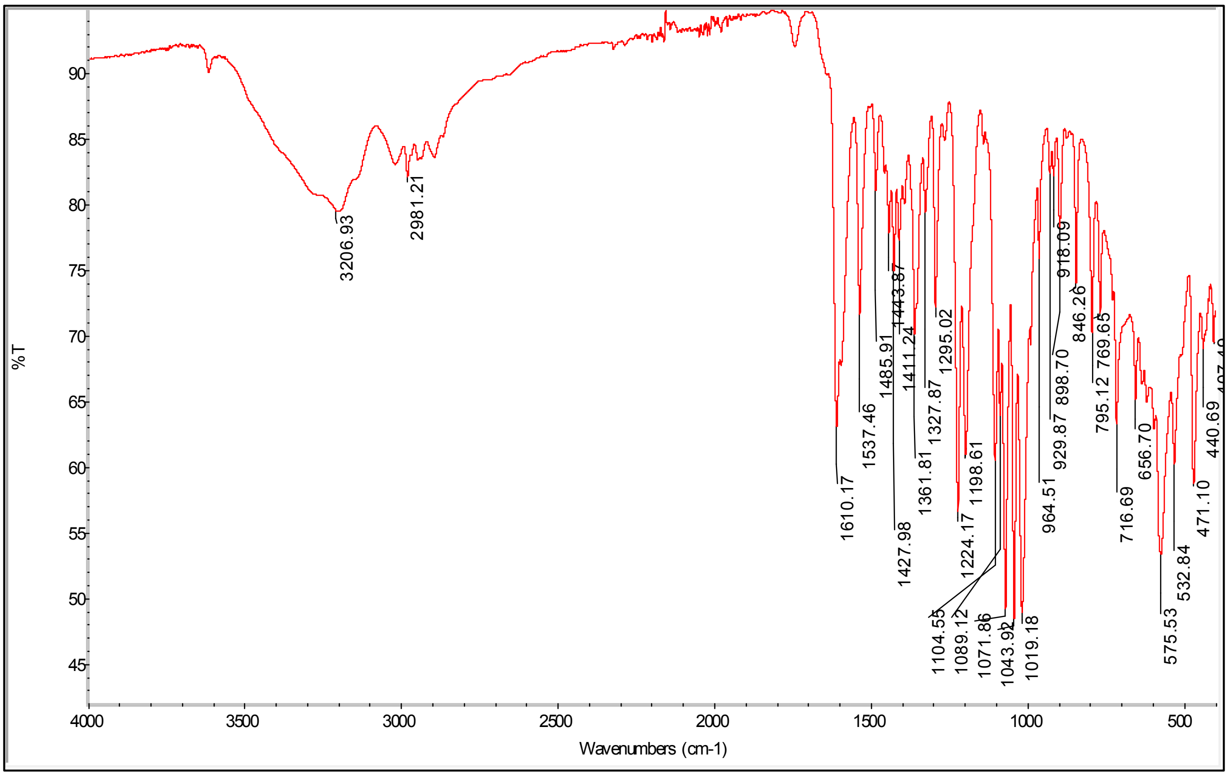


**Figure S24.** ESI-MS (positive ions) for compound **C3** dissolved in methanol.

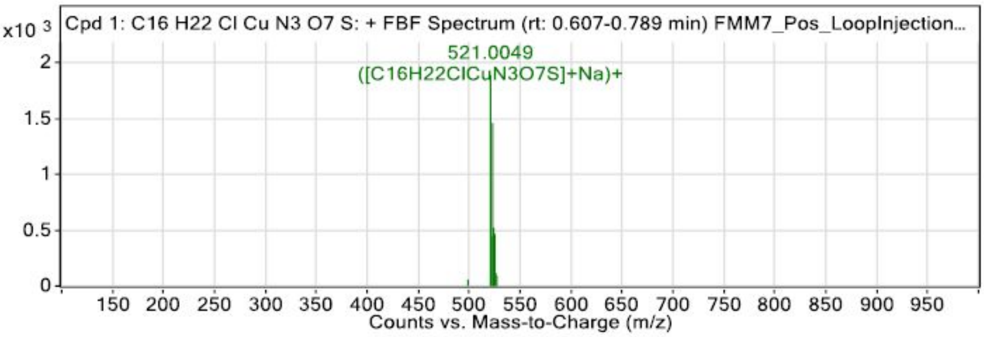

**Figure S25**. ATR-IR of compound **C4**.

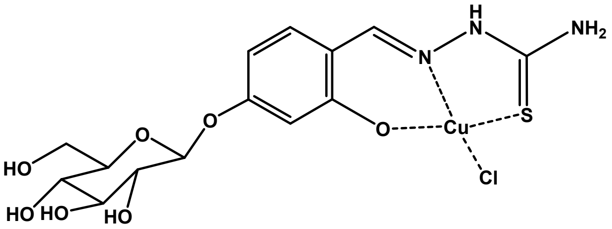


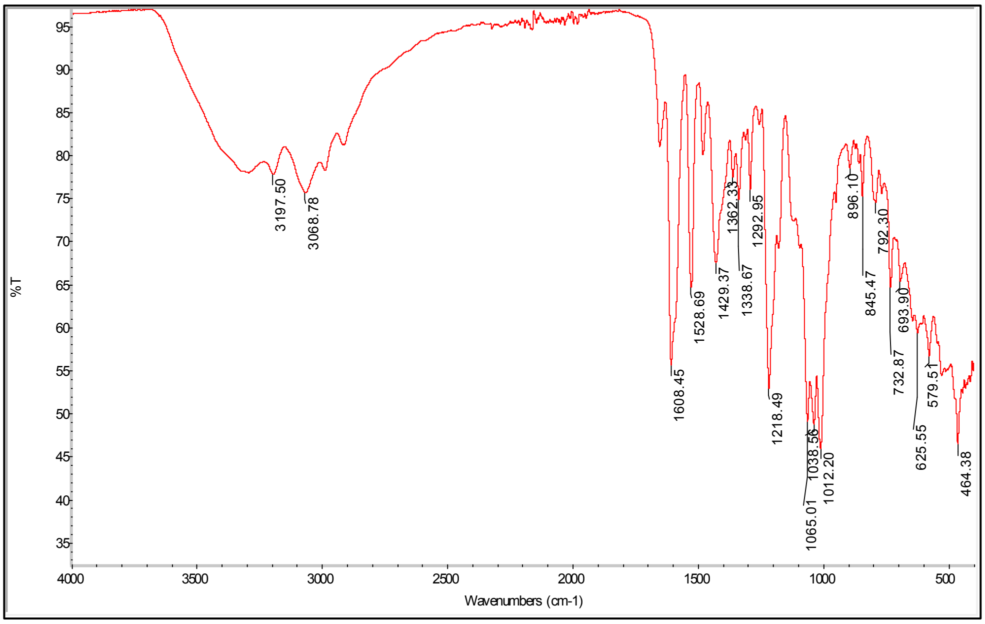


**Figure S26.** ESI-MS (positive ions) for compound **C4** dissolved in methanol.

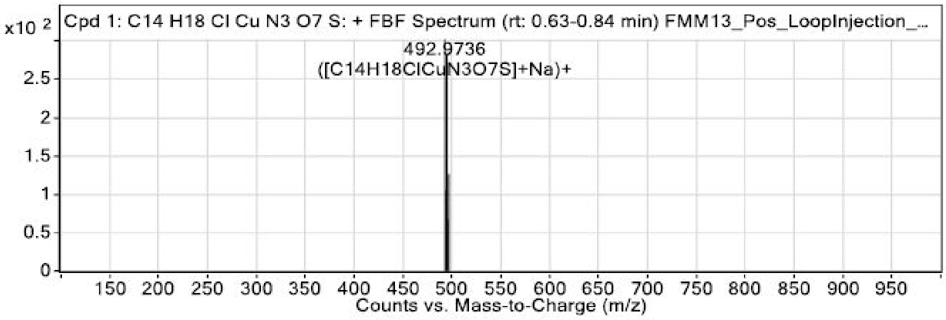

**Figure S27.** Superimposed FT-IR spectra of ligand **$H_2L1$** (red) and the corresponding copper complex **C1** (green).

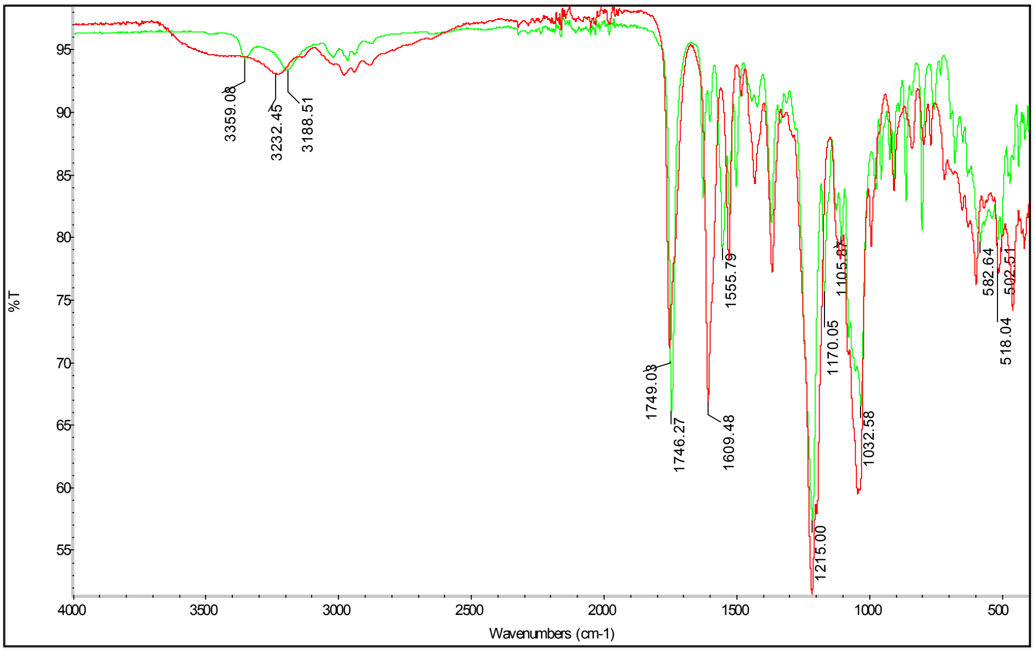

**Figure S28.** UV spectra of **C1-C4** (C ~40-50 μM in 25 mM HEPES buffer, pH = 7.4 in $H_2O$ containing 0.9% NaCl) over 72 hours; the final solutions of C1 and C2 contained 2% DMSO, as these complexes are not completely water-soluble.

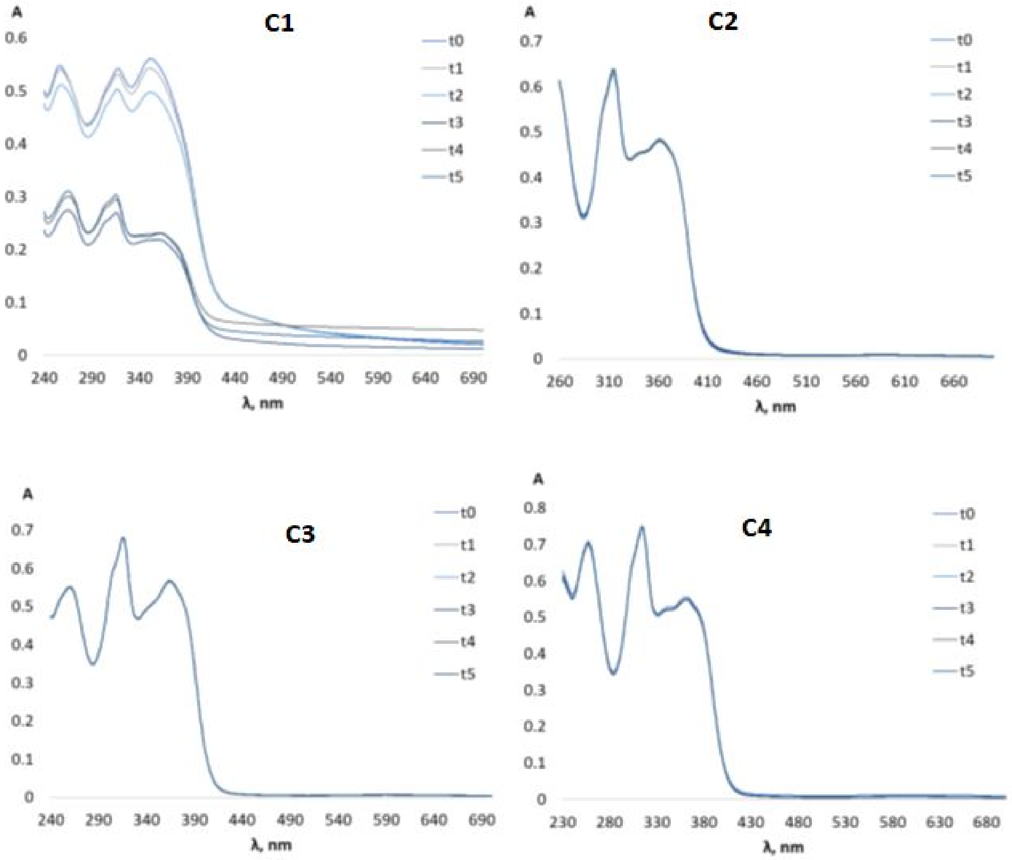

Supplement: Supplementary file 1 [file ao6c02319_si_001.pdf]
